# Mechanism of cell death and its application in the repair of inflammatory bowel disease by mesenchymal stem cells

**DOI:** 10.3389/fimmu.2025.1597462

**Published:** 2025-06-04

**Authors:** Francis Atim Akanyibah, Chang’e He, Peipei Cai, Xiu Wang, Ying Wang, Fei Mao

**Affiliations:** ^1^ Key Laboratory of Medical Science and Laboratory Medicine of Jiangsu Province, School of Medicine, Jiangsu University, Zhenjiang, Jiangsu, China; ^2^ Institute of Hematology, Jiangsu University, Zhenjiang, Jiangsu, China; ^3^ The People’s Hospital of Danyang, Affiliated Danyang Hospital of Nantong University, Zhenjiang, Jiangsu, China

**Keywords:** cell death, extracellular vesicles, exosome, IBD, MSc

## Abstract

The onset and progression of inflammatory bowel disease (IBD), which encompasses ulcerative colitis and Crohn’s disease, are influenced by the immune system, environmental factors, genetics, and intestinal flora. Cell death is a biological phenomenon that occurs in all living organisms; nevertheless, excessive cell death has been linked to IBD, including increased immune and intestinal epithelial cell death and intestinal barrier abnormalities. Anti-tumor necrosis factor medication, which has made significant progress in treating IBD cell death, may fail in some individuals or lose effectiveness over time, necessitating the search for a safe and effective treatment. One of the novel and emerging areas in regenerative and nanomedicine used to regulate cell death is mesenchymal stem cells (MSCs) and their mediators (extracellular vesicles). MSCs and their mediators have been found to attenuate cell death in several illnesses, including IBD. This review explores cell death mechanisms and their implications in IBD, focusing on the potential ameliorative effects of MSCs and their mediators on cell death.

## 1 Introduction

Inflammatory bowel disease (IBD), which encompasses Crohn’s disease (CD) and ulcerative colitis (UC), is defined by persistent inflammation of the gastrointestinal system (1). The immune system, the environment, genetics, and gut microbiota all play a role in the onset and progression of IBD (2). The clinical symptoms in patients with IBD include weight loss, abdominal pain, diarrhea, weakness, blood in the stool, urgent bowel movements, and mucus in the stool (3). Since 1990, the incidence has risen in newly industrialized countries in Africa, Asia, and South America, notably Brazil (4). The burden of IBD is predicted to increase by 2050 due to population expansion and ageing, emphasizing how urgent it is to address the changing public health dilemma that IBD poses (5).

An important biological mechanism for all living creatures is cell death (6). *In vivo*, cell death leads to an inflammatory reaction (7). The ongoing hyperemia, plasma protein leakage, and white blood cell recruitment can be helpful for tissue healing and defense against pathogens (7). This reaction, however, could potentially damage tissue and contribute to the development of certain diseases (7). The emergence of IBDs in both humans and mice has been attributed to cell death mechanisms (8). Apoptosis, pyroptosis, autophagy, ferroptosis, necroptosis, and neutrophil extracellular traps are typical of programmed cell death (PCD) mechanisms (9). These mechanisms are integral to the pathophysiology of IBD, as they lead to intestinal epithelial and immunological cell death (9). Other cell death forms include paraptosis (10), NETosis (11), immunogenic cell death (12), autosis (13), alkaliptosis (14), oxeiptosis (15), erebosis (15), mitoptosis (16), methuosis (17), cuproptosis (18), PANoptosis (19), and entosis (20). Anti-tumor necrosis factor-alpha (TNF-α) medication is a significant breakthrough in IBD treatment, perhaps facilitating mucosal repair by mitigating elevated inflammation-related intestinal epithelial cell (IEC) death (21). On the other hand, some patients either do not react to anti-TNF therapy at all or their response diminishes with time (22). Therefore, comprehending the biology and ramifications of cell death in the intestinal epithelium is essential for developing novel strategies for IBD treatment (21). Furthermore, developing effective medicines to modulate immunological and IEC death pathways can help decrease the growing burden of IBD until 2050.

Mesenchymal stem cells (MSCs) demonstrate a broad range of therapeutic potential in treating IBD (23). MSCs are multipotent stem cells capable of self-renewal and possess various immunomodulatory properties, making them a promising option for treating IBDs (24). The sources of MSCs include dental tissues, menstrual blood, bone marrow, adipose tissue, endometrial polyps, and umbilical cord tissue (25, 26). Researchers have also discovered exosomes in MSCs derived from bone marrow (27, 28), adipose tissue (28, 29), dental pulp (28), menstrual blood (30), and the human umbilical cord (31). According to a recent study, exosomes are significant mediators of MSC function (32). Research shows that MSCs and their exosomes attenuate pyroptosis (31), apoptosis (33), and ferroptosis (34) in dextran sulfate sodium (DSS)-induced IBD. Thus, we review the cell death processes, such as apoptosis, pyroptosis, necroptosis, autophagy, and ferroptosis, and their role in IBD. We also review the potential of MSCs and their extracellular vesicle regulation in cell death to reduce IBD.

## 2 Cell death mechanisms and their implications in IBD

### 2.1 Apoptosis

TNF-related apoptosis-inducing ligand (TRAIL) initiates the extrinsic apoptotic cascade by forming the death-inducing signaling complex (DISC) and activating effector caspases (35). Only death receptors (DR4 and DR5) induce apoptotic signaling among the many TRAIL receptors (35). Tumor necrosis factor receptor 1 (TNFR1) and fas (APO-1/CD95) initiate apoptosis by recruiting caspase-8 via the adaptor fas-associated death domain protein (FADD) (36). Fas directly binds FADD, while TNFR1 indirectly binds FADD through TNF receptor-associated death domain protein (TRADD) (36). TRADD additionally incorporates the RIP-NF-kappaB-inducing adapter (36). Thus, apoptosis communication via death receptors necessitates the acquisition of adaptor proteins (TRADD and FADD) and caspase-8 and caspase-10, which might serve comparable roles in apoptosis onset (37–40). There are two successive signaling networks involved in TNFR1-mediated apoptosis. The first plasma membrane-bound complex (complex I) comprises TNF receptor-associated factor 2 (TRAF2), receptor-interacting protein kinase(RIP1 kinase/RIPK1), TRADD, and TNFR1, which triggers NF-kappa B activation (39). Subsequently, FADD, caspase-8, TRADD, and RIP1 unite to create a cytoplasmic complex known as complex II (39).

BAK and BAX are proteins that trigger mitochondrial membrane permeabilization, releasing cytochrome C and activating apoptotic caspases, thereby facilitating mitochondrial apoptosis (41). BAK and BAX are essential regulators of apoptosis that mediate the critical process of permeabilization of the outer membrane of mitochondria (42). The recognized procurers of the death signal in this segment of the apoptotic cascade are caspase-8 and BID (43). The proapoptotic family members BAK or BAX oligomerize in response to activation of BID, a “BH3-domain-only” BCL-2 family member, releasing mitochondrial proteins into the cytosol (44). The activation of BAX/BAK is indirectly triggered by the deactivation of anti-apoptotic BCL-2 proteins by BH3-only proteins (45). Cytochrome C is crucial for activating the apoptotic intrinsic pathway, which triggers the caspase cascade by connecting to apoptotic protease activating factor-1 (APAF-1) (46). Cytochrome C release causes APAF-1 to oligomerize, forming the huge complex known as an apoptosome (47). The apoptosome recruits and activates procaspase-9, which then triggers caspase-3 processing downstream (47). These processes are characteristics of the intrinsic pathway.

**Figure 1:**
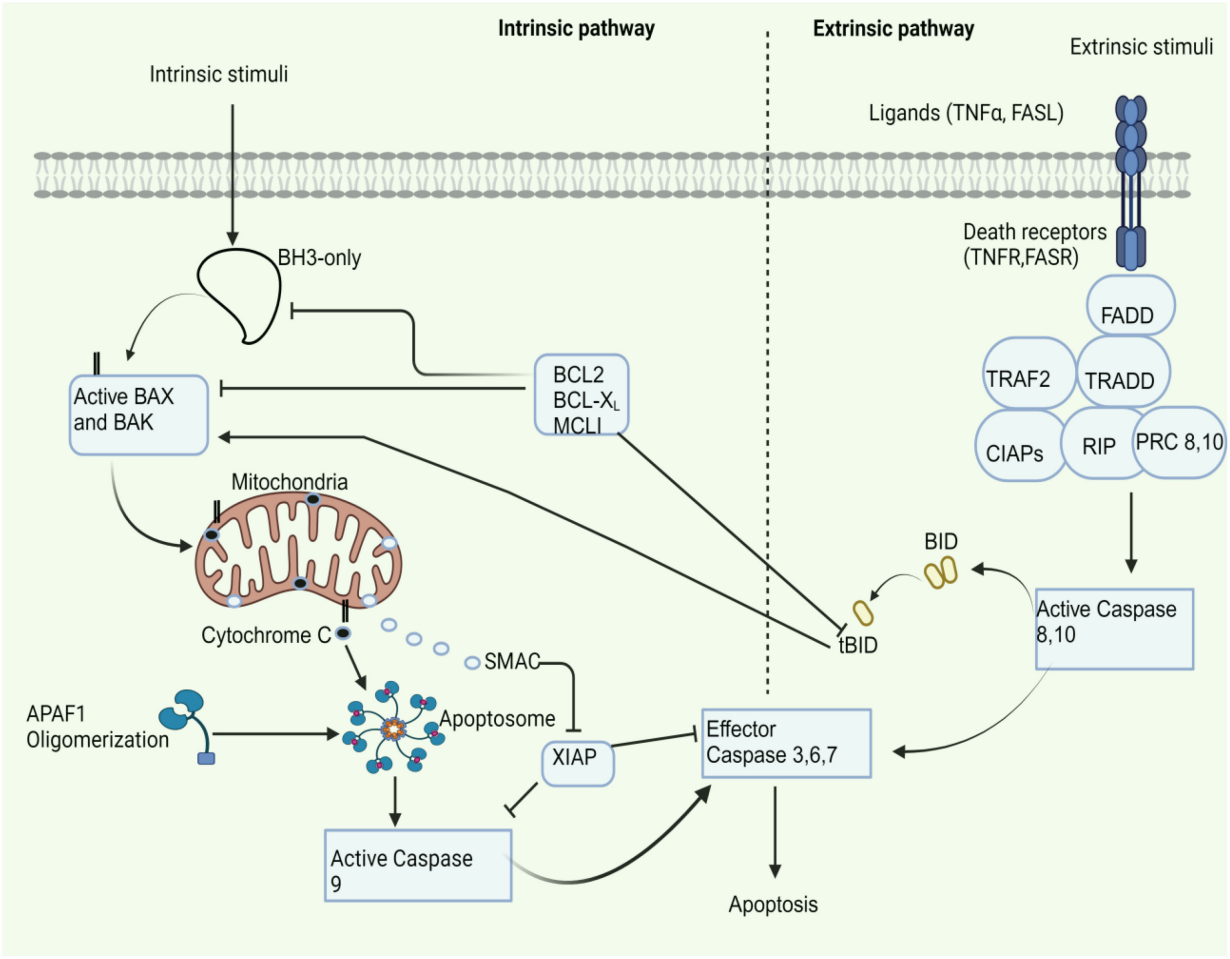
Apoptosis pathway. Intrinsic stimuli like hypoxia, DNA damage, and ER stress all contribute to this pathway. Extrinsic stimuli like TNF alpha and FASL contribute to the extrinsic pathway. Ultimately, both routes activate the effector caspases, resulting in apoptosis. APAF1, apoptotic protease activating factor-1; BAX, bcl-2 associated x protein; BCL2, b-cell lymphoma-2; BH3, bcl2 homology domain 3; CIAPs, cellular inhibitor of apoptosis proteins; FADD, fas-associated death domain protein; FASL/R, Fas ligand/receptor; PRC: procaspase; SMAC, second mitochondrial-derived activator of caspase; tBid, truncated bid; TNFα/R, tumor necrosis alpha/receptor; TRADD, TNF receptor-associated death domain protein; TRAF2, TNF receptor-associated factor 2; XIAP, x-linked inhibitor of apoptosis protein.

During the last stages of apoptosis, caspases-3,-6, and -7 are activated by the extrinsic (mediated by caspase-8) and intrinsic pathways (mediated by caspase-9), facilitating the cleavage of additional proteins (8). A powerful inhibitor of caspases 3, 7, and 9 is an x-linked inhibitor of apoptosis protein (XIAP). During apoptosis, the release of mitochondrial SMAC (the second mitochondrial-derived activator of caspase) suppresses XIAP activity (48). **Figure 1** shows the intrinsic and extrinsic pathways of apoptosis. BCL-2 and BCL-XL do not affect TRAIL-induced apoptosis in lymphoid cells, but they can prevent or delay apoptosis in nonlymphoid cancer cells (49). BCL-XL and antioxidant enzymes prevent mitochondrial cytochrome C release and reactive oxygen species (ROS) formation in a cell-free reconstitution system caused by caspase-8-mediated BID cleavage and recombinant truncated Bid (tBid) (50).

#### 2.1.1 Apoptosis in IBD

##### 2.1.1.1 Apoptosis molecule expressions in IECs

Studies show higher levels of apoptotic molecules in the IEC, suggesting they may be implicated in the pathophysiology of IBD. For instance, current research found that IECs of DSS-induced animals produce more pro-apoptotic proteins (BAX and cleaved caspase-3) and fewer anti-apoptotic proteins (BCL2) (51). Similarly, Zhang and the team found that TNBS-treated mice’s IECs expressed more BAX and caspase 3 and less BCL2 (52). Also, Li and colleagues observed that mice given TNBS show increased caspase 3 and BAX expression in their IECs while reducing BCL2 (53). In a different study, only BCL-XL, one of the anti-apoptotic BCL2 proteins, is significantly elevated in human CRC tissues (54). After *adenomatous polyposis coli* (*APC*) loss, BCL2 is necessary for effective intestinal transformation and may be a target for chemoprevention (55). In acute lymphoblastic leukemia, elevated BCL2 expression has been noted (56). This may suggest that whereas BCL2/BCL-XL decreases in IBD, it is high in cancer; hence, elevated BCL2/BCL-XL may be required for tumor growth in cancer.

Clinical investigations have shown that apoptosis regulators can induce apoptosis in IECs. These regulators increase in patients with IBD. For instance, p53-upregulated modulator of apoptosis (PUMA) expression was found to be higher in colitis-affected tissues in UC patient samples, and it is linked to apoptotic induction and colitis severity. PUMA activation promotes IEC apoptosis, which aids in the pathophysiology of colitis (57). Dirisina et al. (58) found that in patients with UC, levels of p53 and PUMA are elevated in inflamed mucosal tissues. This suggests human colon inflammation activates IEC apoptosis through p53-independent and p53-dependent pathways. Additionally, PUMA triggers an intrinsic apoptosis pathway associated with colitis.

##### 2.1.1.2 Apoptosis, immune cells and intestinal barrier integrity

In crypts of affected and nearby uninvolved regions, apoptosis is the primary cause of epithelial cell death in active UC, with the Fas/Fas-L relationship acting as a mediator (59). Thus, investigations have revealed that the Fas/Fas-L connection may be present in immune cells, promoting apoptosis in IBD. For instance, a study revealed that FasL is present in CD3 lymphocytes penetrating UC lesions, suggesting Fas-FasL-induced apoptosis contributes to UC mucosal injury (60). According to separate research, immune cells such as T cells and macrophages may be less likely to suffer apoptosis when activated by Fas-FasL interaction. IBD patients with reduced Fas expression on intestinal lamina propria (LP) T-cells and macrophages may have a reduced susceptibility to Fas/FasL-mediated apoptosis (61). This suggests that higher Fas expression on LP T cells may enhance sensitivity to Fas/FasL apoptosis. Interestingly, the interstitial CD95L+ cell count and apoptosis frequency in the LP and epithelium are significantly elevated in UC. Subepithelial CD95L+ mononuclear cells are shown to be focally associated with epithelial apoptosis (62). *In vitro* studies indicate that activated T cells are the primary source of CD95L expression, suggesting that CD95L regulates immunological responses (63). As a result, T cells may be activated, increasing CD95L+ cells/mononuclear cells in the LP and epithelium.

Downregulation of specific proteins/enzymes has also been demonstrated to disrupt the intestinal barrier, resulting in inflammation and elevated apoptotic genes. A recent study found that downregulation of CRL4^DCAF2^ in IECs results in gut barrier dysfunction and inhibits IEC growth, increasing its susceptibility to inflammation. Inflamed colon tissues of mice lacking DCAF2 exhibited elevated levels of cleaved caspase 3 and other genes like p53, BAX, and Bid (64). Another study also found that the knockdown of 3-mercaptopyruvate sulfurtransferase significantly increased the expression of cleaved caspase 3 and 8, decreased BCL-XL, and enhanced the experimental colitis induced by DSS. Intestinal epithelial damage and a ruptured barrier were also found (65). Zhang et al. (66) also found that the knockout of DJ-1 in mice significantly exacerbated colitis, resulting in increased intestinal inflammation and worsened IEC apoptosis. DJ-1−/− mice showed significantly higher cleaved, activated forms of caspase 3 and caspase 7 levels after DSS treatment than wild-type mice.

##### 2.1.1.3 Apoptosis and the gut microbiome

The microbiota can either promote intestinal epithelial integrity or cause mucosal inflammation by causing or preventing intestinal epithelial cells from undergoing apoptosis (67). Therefore, studies have shown that the gut microbiota can induce apoptosis in the IEC. A study indicates that *Cryptosporidium parvum* (*C. parvum*) causes moderate apoptosis in human IECs, with the highest levels occurring 24 hours after infection (68). A recent study has shown that miR-3976, which targets BCL2A1, regulates cell apoptosis and parasite load in HCT-8 cells after *C. parvum* infection (69). This further reveals the role of *C. parvum* in regulating apoptosis in IECs.

## 3 Pyroptosis

Pyroptosis is an inflammatory PCD process triggered by mouse caspase-11, human caspase-4 and 5, or both human and mouse caspase-1 (70). Pyroptosis can be either canonical or noncanonical. The canonical pathway reacts to pathogen-associated molecular patterns (PAMPs) and damage-associated molecular patterns (DAMPs) during microbe infection, and the noncanonical pathway reacts to Gram-negative bacteria’s internal lipopolysaccharides (LPS) (71). In both pathways, pyroptosis occurs when inflammatory caspases cleave and activate the pore-forming effector protein gasdermin d (GSDMD) (70).

Canonical inflammasomes are produced by cytosolic pattern-recognition receptors (PRRs) in the presence of pathogen-related signals. This process activates pro-caspase-1 and results in pyroptotic cell death (72). The adaptor protein apoptosis-associated speck-like protein containing CARD (ASC) oligomerizes with inflammasome-forming PRRs, creating a massive cytosol structure that triggers dimerization, autoproteolysis, and pro-caspase-1 zymogen activation (72). A study found that cigarette smoke extracts (CSE) increase NOD-like receptor pyrin domain-containing protein 3 (NLRP3) and caspase-1 activity levels and improve the release of IL-1β and IL-18 in 16 bronchial epithelial cells (73). However, a recent study found that CSE enhances pro-IL-1β expression, activates caspase-1, and releases IL-1β and IL-18 without NLRP3 (74), implying that activation of caspase 1 may either be dependent or independent of NLRP3.

In the noncanonical pathway, Yang and the team found that LPS activation in the cytosol triggers caspase-11-dependent cleavage of the pannexin-1 channel, followed by ATP release, activating the purinergic P2X7 receptor, leading to cytotoxicity. These pathways are essential for initiating endotoxic shock in mice. The study finally found that caspase-11’s noncanonical inflammasome pathway triggers the pannexin-1 channel, leading to K+ efflux and NLRP3 activation (75). In response to microbial infection and cellular injury, the NLRP3 inflammasome plays a crucial role in the innate immune system by mediating caspase-1 activation and the release of proinflammatory cytokines, IL-1β, and IL-18 (76).

**Figure 2:**
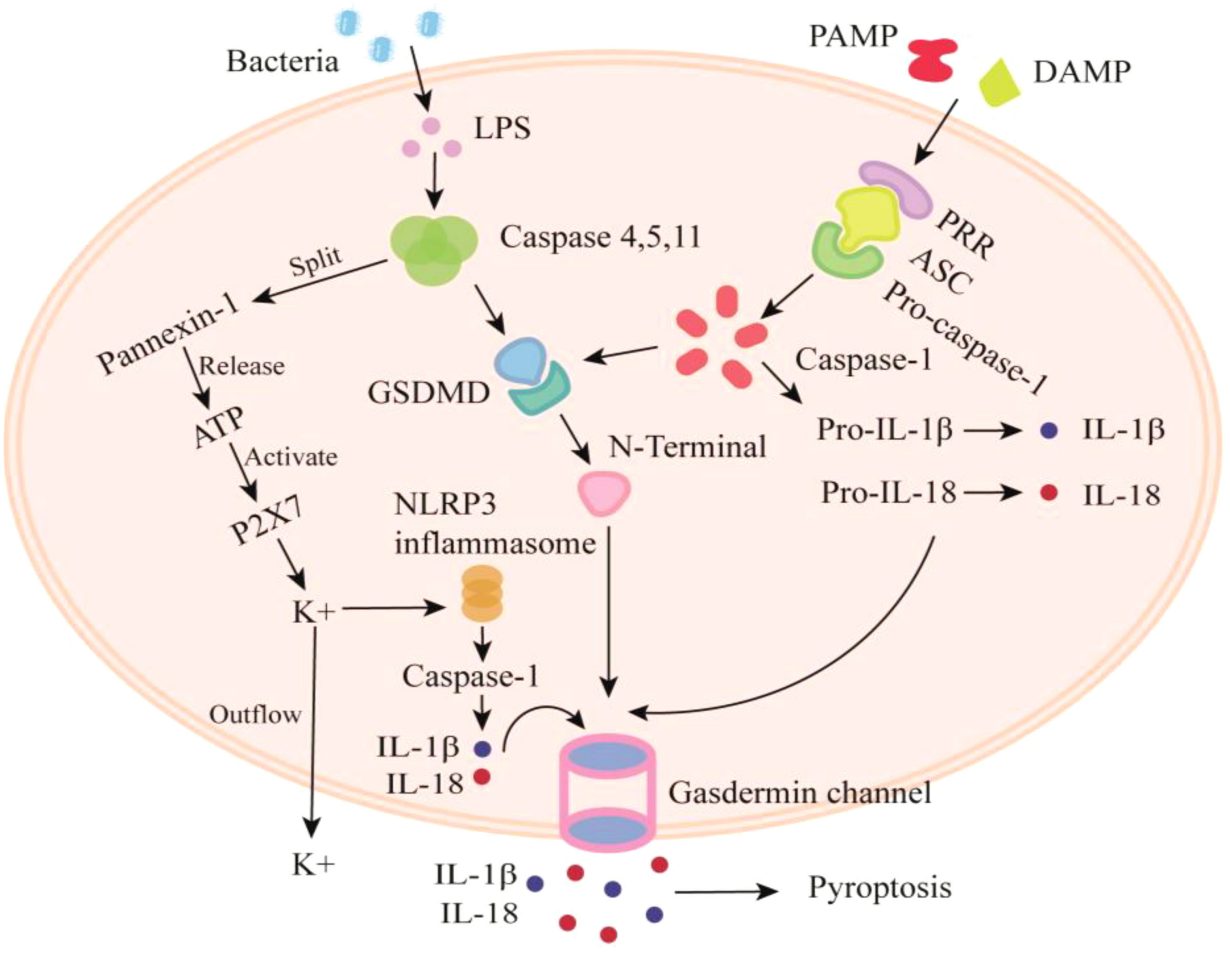
Mechanism of pyroptosis. PAMP and DAMP commence the canonical route, whereas bacteria and LPS synthesis initiate the non-canonical route. These two routes eventually cleave GSDMD, resulting in the GSDMD channel that leaks IL-1β and IL-18, causing pyroptosis. ASC, apoptosis-associated speck-like protein containing card; ATP, adenosine triphosphate; DAMP: damage-associated molecular patterns; GSDMD, gasdermin d; IL, interleukin; NLRP3: nod-like receptor pyrin domain-containing protein 3; PRR, pattern-recognition receptor; PAMP, pathogen-associated molecular patterns.

Pyroptosis’ final stage requires caspase 1 in the conventional pathway and caspase 4/5/11 (caspase 4/5 in humans, caspase 11 in mice) in the noncanonical pathway to cleave GSDMD at D275 into N- and C-termini. After cleavage, GSDMD’s N-terminus generates a transmembrane pore that leaks cytokines like IL-1β and IL-18 and messes with the water and ion balance, leading to severe inflammation and eventual cell death (71). **Figure 2** depicts the mechanism of pyroptosis.

### 3.1 Pyroptosis in IBD

#### 3.1.1 Intestinal epithelial barrier

Tight junction proteins, including occludins, zonula occludens (ZO), and claudins, are crucial for preserving the epithelial barrier integrity (77). Therefore, pyroptosis can weaken the intestinal barrier’s integrity by reducing tight junction proteins. A study found that mice treated with 5% DSS experienced mucin and occludin loss, increased inflammation, and NLRP3-related protein expression. The DSS group also showed increased levels of ASC, caspase-1, GSDMD, and IL-1β with decreased occludin protein levels (78). Similarly, Mahmoud and team also found increased IL-1β levels and elevated NLRP3, cleaved caspase-1, and ASC expressions in the colon tissues of DSS-induced mice. Additionally, DSS treatment decreased occludin gene expression and claudin-1 expression in immunohistochemistry (IHC) (79). Another study found that SP23 administration reduces ZO-1, occludin, and claudin-1 loss and downregulates STING and NLRP3 signaling pathways in intestinal inflammation caused by DSS (80). The study suggests that an increase in NLRP3 may lead to a decrease in tight junction proteins in DSS.

#### 3.1.2 Pyroptosis and immune cells

*In vitro*, macrophages cultured with DSS secrete high amounts of IL-1β in a caspase-1-dependent manner (81). Macrophages lacking ASC, NLRP3, or caspase-1 show reduced IL-1β production, suggesting that DSS triggers caspase-1 via the NLRP3 inflammasome (81). Another study also found that Galectin-3 expression contributes to acute DSS-induced colitis by activating the NLRP3 inflammasome and producing IL-1β in macrophages (82). Liu and the team revealed that salidroside skews macrophage pyroptosis and T helper 17 (Th17)/Treg balance to protect against experimental colitis (83). V-set and immunoglobulin domain-containing 4 (VSIG4), a type I transmembrane receptor found in tissue-resident macrophages, has been shown to have anti-inflammatory effects on immune-related illnesses (84). Liao and team found that VSIG4 is downregulated in IBD and negatively correlates with the NLRP3 inflammasome. The study reveals that M1 macrophages exhibit a greater NLRP3 inflammasome, pyroptosis, and inflammatory response than M2 macrophages (84). The study suggests that M1 macrophages may be involved in pyroptosis, while M2 may suppress it. In an alternative investigation, Shi and colleagues found that in macrophages, REGγ inhibition regulates members of the gasdermin family, promoting pyroptosis (85). Blocking REGγ can induce pyroptosis in macrophages.

A type of T cell and enzyme deletion has been shown to induce pyroptosis in tumor cells and IBD, respectively. For instance, Le Floch and team found that Vγ9Vδ2 T cells stimulated by 107G3B5 trigger caspase 3/7, leading to tumor cell death by pyroptosis. Therefore, using 107G3B5 to target BTN2A1 increases the Vγ9Vδ2 T-cell antitumor response by inducing immunogenic cell death through pyroptosis (86). Also, *methyltransferase-like 13* (*METTL3)* deletion in IBD increases colonic epithelial cells’ vulnerability to pyroptosis and abnormal CD4+ T cell proliferation (87).

#### 3.1.3 Other triggers of pyroptosis

Ma and the team found that long-term light exposure causes intestinal inflammation, which is linked to the gut microbiota and NLRP3 inflammasome activation. The study further found that the activation of the NLRP3 inflammasome is positively connected with gut microbiota dysbiosis. *Bifidobacterium* and *unclassified Oscillospirales* relative abundances were positively connected with *NLRP3* mRNA expression levels. Additionally, the amount of *caspase-1* and *IL-1β* mRNA expression was positively connected with the relative abundance of *Family_XIII_UCG-001 *(88).

Zhang and the team found that miR-223 promotes cell pyroptosis and contributes to the pathophysiology of IBD by activating the NF-κB pathway by targeting smad nuclear-interacting protein 1. Pyroptosis was reduced when miR-223 was knocked down. The IBD cell model’s ASC, NLRP3, and caspase-1/pro-caspase-1 levels were considerably lowered by miR-223 downregulation (89).

#### 3.1.4 Pyroptosis markers in clinical study

Research in clinical settings has indicated that markers of pyroptosis, such as NLRP3 and its downstream pro-inflammatory cytokines, are present in patients with IBD. This leads to the release of proinflammatory cytokines such as IL-1β and IL-18. For instance, Lazaridis and the team found that the NLRP3 inflammasome is active in CD patients. NLRP3 activation is only observed in UC patients with a long-standing history of the disease. CD patients exhibited a significantly higher mean maximal percentage increase in IL-1β release than controls and UC patients (90). Similarly, it was discovered that active UC and CD had higher levels of *NLRP3* and *IL-1β* (91). However, NLRP3 and IL-1β were found in active UC, a neutrophil-dominated lamina propria cell population, indicating that IL-1β is processed independently of the inflammasome (91). Another study examined the potential link between NLRP3 and IBD in the Chinese Han community. It was discovered that the Chinese Han population’s NLRP3 polymorphisms rs10754558 and rs10925019 are strongly linked to UC susceptibility but not CD, suggesting NLRP3 may be crucial to UC pathophysiology (92).

CARD8 is a negative regulator of NLRP3 (93). It has been found that patients with missense mutation CARD8 CD have higher IL-1β levels than healthy controls, and when peripheral monocytes are stimulated with NLRP3 activators, they produce more IL-1β. The mutant T60 CARD8 could not bind to NLRP3 and prevent its oligomerization, undermining the NLRP3 inflammasome (94). This suggests that active NLRP3, a marker of pyroptosis, may be linked to CD patients.

#### 3.1.5 Pyroptosis-related genes as biomarkers

Zhao and colleagues discovered potential genes such as *AIM2*, *ZBP1, CASP1, IL1β, CASP11*, and *TLR4* in UC and *CASP11*, and *TLR4* in active UC. To identify genes, the researchers employed a variety of analytical approaches such as binary logistic regression, least absolute shrinkage and selection operator, random forest analysis, and artificial neural networks. Ultimately, the study found that these genes function well in differentiating between UC and determining if the condition is active (95). Another study found that *AIM2* expression level could be a biomarker for predicting anti-TNF therapy efficacy. The hub genes found in the study were CAPS1, CASP5, GSDMD, AIM2, and NLRP3, with AIM2 being the best predictor of anti-TNF medication response. Immune function was greater, and anti-TNF medication was less effective in patients with a larger burden of the AIM2 inflammasome (96). A different research also identified *ZBP1* and *AIM2* as genes related to PANoptosis in atherosclerosis (97). Deep and machine-learning models have significantly improved the efficiency of IBD diagnosis and assessment by automating the accurate analysis of data from various diagnostic modalities (98). These techniques significantly reduce physicians’ time manually reviewing data for evaluation (98).

## 4 Necroptosis

Necroptosis, like other cell deaths such as apoptosis and necrosis, is a caspase-independent PCD mechanism that is believed to be a significant factor in the etiology of various illnesses (99). These include inflammatory conditions of the gut, skin, and lungs, in addition to kidney, heart, and brain ischemia-reperfusion injuries (100). The basic machinery that drives the route consists of the RIPK1 and RIPK3 kinases and the terminal effector pseudokinase mixed lineage kinase domain-like (MLKL), which combine to create cytoplasmic necrosomes, leading to cell enlargement, plasma membrane rupture, intracellular component leakage, and cellular death and inflammation development (101, 102).

The canonical and noncanonical pathways make up this process. In the canonical pathway, it has been shown that RIPK1, RIPK3, and MLKL are the essential components when caspase-8 is deficient or inhibited (103). Necroptosis happens when the caspase activity necessary for apoptosis is inhibited in response to TNF, Fas, or TRAIL, as well as certain toll-like receptor (TLR) ligands (104). The primary mechanism of how necroptosis commences is the liberation of RIPK3 from caspase-8-induced repression (105). Also, Interferon (IFN)-β-induced macrophage necroptosis is triggered by tonic IFN-stimulated gene factor 3 (ISGF3) signaling, resulting in the sustained expression of signal transducer and activator of transcription (STAT)1, STAT2, and interferon regulatory factor 9 (IRF9) (106). Type I (mostly α/β) and type II (γ) IFNs both trigger pro-necrotic signaling through transcriptional activation of the latent kinase PKR that is dependent on Janus kinase (JAK)/STAT (107). Type I and type II IFNs trigger RIPK1/3 kinase-mediated necrosis when caspases (e.g., caspase 8) are inactivated or FADD is deleted or rendered inactive by phosphorylation (107).

**Figure 3:**
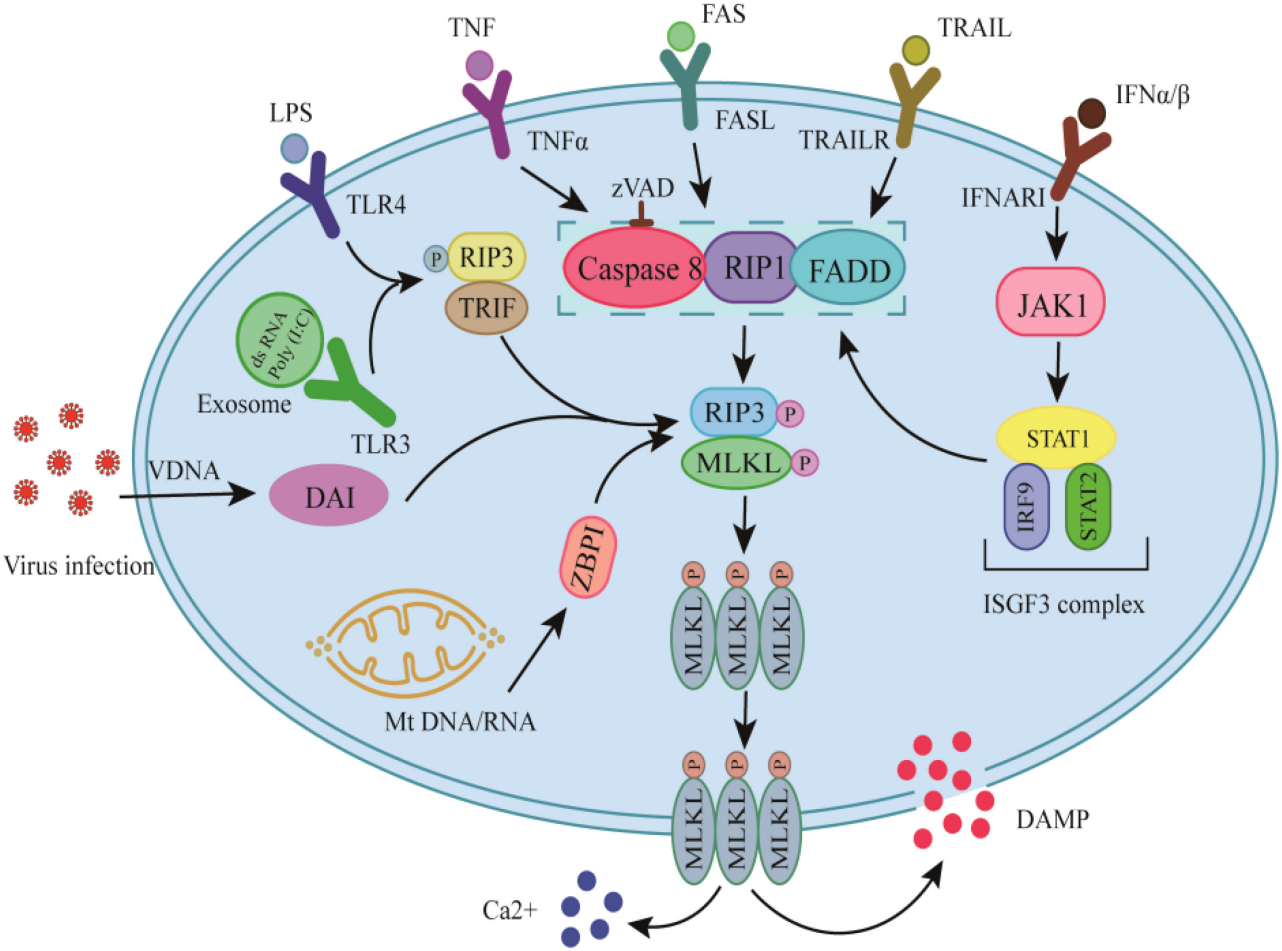
Necroptosis mechanism. The canonical and non-canonical paths lead to RIP3/MLKL activation, triggering the oligomerization of MLKL, resulting in the rupture of the plasma membrane, the release of intracellular chemicals, and cell death and inflammation promotion. FADD, fas-associated death domain protein; IFN, Interferon;IFNARI, ifn-α receptor type I; IRF9, interferon regulatory factor 9; ISGF3, ifn-stimulated gene factor 3; JAK1, janus kinase 1; LPS, lipopolysaccharides; MLKL, mixed lineage kinase domain-like; mt, mitochondrial; RIP/RIPK, receptor-interacting protein kinase; STAT, signal transducer and activator of transcription; TLR3/4, toll-like receptor-3/4; TNFα, tumor necrosis factor (TNF) alpha; TRAIL/R, TNF-related apoptosis-inducing ligand/receptor; TRIF, TIR domain-containing adapter-inducing interferon-β; ZBP1 (DAI), Z-DNA binding protein 1; VDNA, DNA viruses.

The non-canonical route may not require the presence of all the kinases, such as RIPK1/3 and MLKL. For instance, the mouse cytomegalovirus infection results in necrosis that is RIPK3-dependent (108). Another study found that Influenza A virus (IAV) replication triggers Z-RNAs to activate ZBP1 in the nucleus, initiating RIPK3-mediated MLKL activation, leading to nuclear envelope rupture, DNA leakage, and necroptosis (109). Also, ZBP1 triggers RIPK3/MLKL signaling upon detecting cytosolic mitochondrial (mt)DNA (110). A recent study has shown that IAV-induced necroptosis requires RIPK3 in epithelial cells (111). These show that some viral infections may trigger necroptosis without RIPK1. **Figure 3** depicts the fundamental machinery in these routes.

### 4.1 Necroptosis in IBD

#### 4.1.1 Necroptosis and immune cells

Using confocal scanning, Lee and the team found that the development of UC is linked to CD4+ T cell necroptosis and inflammation. RIPK3, phosphorylated (p)-MLKL, and IL-17A are expressed more in CD4+ T cells in involved tissue from UC patients than in uninvolved tissue (112). Therefore, CD4+ T cell necroptosis may play a role in UC. Brasseit and team also found that colitogenic T lymphocytes are crucial for the initiation and progression of colitis. Colitogenic T-cell depletion decreases TNFα levels and inflammatory immune cell infiltration at inflammation sites (113).

It has been shown that once T-cell immunoglobulin domain and mucin domain-3 (Tim-3) knockdown macrophages attract neutrophils with their released chemokines, they emit TNF-α to cause neutrophil necroptosis. Consequently, this weakens the gut’s mucosal barrier and creates a vicious loop in colitis development (114). This suggests that macrophages may be involved in neutrophil necroptosis, which leads to colitis development.

#### 4.1.2 Necroptosis and intestinal epithelial barrier

The intestinal epithelial barrier is crucial for maintaining host homeostasis (115). The intestinal epithelial barrier, composed of epithelial cells, tight junction proteins, and gut secretions, impedes the movement of antigens and luminal chemicals across the paracellular space (116). The stability of the epithelial barrier depends on tight junction proteins called occludins, claudins, and zonula occludens (77). Therefore, numerous harmful events that disrupt the tight junction complex can result in the loss of this homeostatic barrier (117).

A study by Liu and the team found that necroptosis disrupts the intestinal epithelial barrier by suppressing claudin-1 and occludin. However, Nec-1 inhibited necroptosis, which increased claudin-1 and occludin protein expression (118). Another study also showed that an RIPK1 inhibitor may reduce intestinal barrier damage by reducing tight junction breakdown and the oxidative stress that comes with it (119). This may imply that RIPK1, which is involved in necroptosis in IBD, may damage the intestinal barrier.

Negroni et al. (120) also revealed that necroptosis driven by RIPK3 significantly affects intestinal inflammation by increasing pMLKL, activating various cytokines and alarmins, and modifying epithelial permeability (E-cadherin, Occludin, Zonulin-1). The overexpression of RIPK3 leads to a reduction in the integrity of the intestinal epithelial barrier. In human intestinal epithelial cells, RIP3 inhibitor GSK872 or RIP3 knockdown reverses TNF-α’s promotion of necrosis and apoptosis and its negative influence on proliferation (121). This implies that RIP3 presence may lead to the disruption of the intestinal epithelial barrier.

Zhang et al. (122) found that non-littermate MLKL-deficient mice exhibit considerably better survival rates, clinical scores, intestinal damage, and intestinal mucosal barrier integrity than wild-type (non-littermate) mice. Schwarzer et al. (123) also found that MLKL loss only partially alleviated ileitis in animals lacking FADD in IECs, while it completely cured ileitis caused by epithelial caspase-8 ablation.

Notably, STATI has been demonstrated to cause necroptosis in IBD. Stolzer and team found that in IEC mice lacking caspase-8, an additional loss of STAT1 prevented cell death, barrier disruption, and systemic infection. When epithelial STAT1 is absent, epithelial cells are no longer lost, and caspase-8 activation is also decreased. Both caspase-8-dependent and -independent cell death are upstreamed by epithelial STAT1 (124). Controlling intestinal barrier penetration by beneficial and harmful microorganisms depends on Paneth cells (125). In IBD, it is common to observe a decrease in the number of Paneth cells (126). This suggests that in IBD, barrier breakdown may be caused by Paneth cell loss. Interestingly, IFNL (Interferon lambda) induces Paneth cell death in mice via MLKL and STAT1 activation (127). Also, TNF-α and IFN-γ, Th1-type cytokines, disrupt the gut epithelial barrier function and occupy crucial nodes within these networks. It is found that JAK1/2 kinases are the primary and nonredundant drivers of the synergistic death of human IECs induced by IFN-γ and TNF-α (128). These imply that STAT1/JAK1/2 may induce necroptosis in IEC, resulting in barrier breakdown.

#### 4.1.3 Necroptosis in clinical studies

Necroptosis molecules may cause IBD, according to several clinical investigations. For instance, Duan et al. (121) found that as the severity of UC worsened, the expression levels of MLKL and RIP3 rose considerably. Similarly, in UC’s inflammatory tissues, RIP3 and MLKL are elevated (129). According to the study, intestinal inflammation in UC patients is closely linked to necroptosis (129).

Patients with IBD have higher levels of RIPK3 expression in inflammatory tissues than controls (130). Pierdomenico and the team also found that patients with IBD and allergic colitis have higher levels of RIP3 and MLKL in their inflammatory tissues, although caspase-8 was lower. Children with IBD have intestinal inflammation that is tightly linked to necroptosis, which exacerbates the inflammatory process (105).

## 5 Autophagy

Cells use autophagy to break down and recycle proteins and organelles to preserve intracellular homeostasis. Autophagy generally protects cells; nevertheless, excessive autophagic flux or disruption of autophagy pathways typically results in cell death (131).

AMP-activated protein kinase (AMPK), a crucial energy sensor that controls cellular metabolism to preserve energy homeostasis, stimulates autophagy. Nevertheless, the mammalian target of rapamycin (mTOR), a major regulator of cell development that combines signals from growth factors and nutrients, inhibits autophagy (132). AMPK phosphorylates Ser 317 and Ser 777 of unc-51-like kinase 1 (ULK1) in response to a glucose shortage, hence inducing autophagy. High mTOR activity inhibits ULK1 activation when nutrients are sufficient by phosphorylating ULK1 Ser 757 and disrupting the ULK1-AMPK connection (132). Melatonin has been found to inhibit cancer cells by activating autophagy through ULK1 activation, following mTOR inhibition, which phosphorylates Beclin-1. Beclin-1 stimulates autophagy and phosphatidylinositol (3,4,5)-trisphosphate kinase (PI3K) complex I activity in cancer cells in conjunction with autophagy/beclin-1 regulator 1 (AMBRA1) and vacuolar protein sorting 34 (VPS34) (133). These show that AMPK, beclin-1, PI3K, and VPS34 may be involved in autophagy.

The autophagosome, a crucial initial step in autophagy, is a double-membrane organelle that absorbs cytosolic material for degradation. ULK1 mediates this phase by forming a complex with three protein partners: focal adhesion kinase family interacting protein of 200 kDa (FIP200), autophagy-related protein (ATG) 13, and ATG101 (134). During this process, ATG8 is incorporated into the expanding phagophore via covalent attachment to phosphatidylethanolamine via the noncanonical ubiquitin-like conjugation cascade, including the E1 (ATG7), E2 (ATG3, ATG10), and E3 (ATG12-ATG5-ATG16 complex) enzymes (135). A study reveals that the ATG8 conjugation machinery, consisting of six ATG proteins, regulates the shape of the membrane during autophagosome development (136).

**Figure 4:**
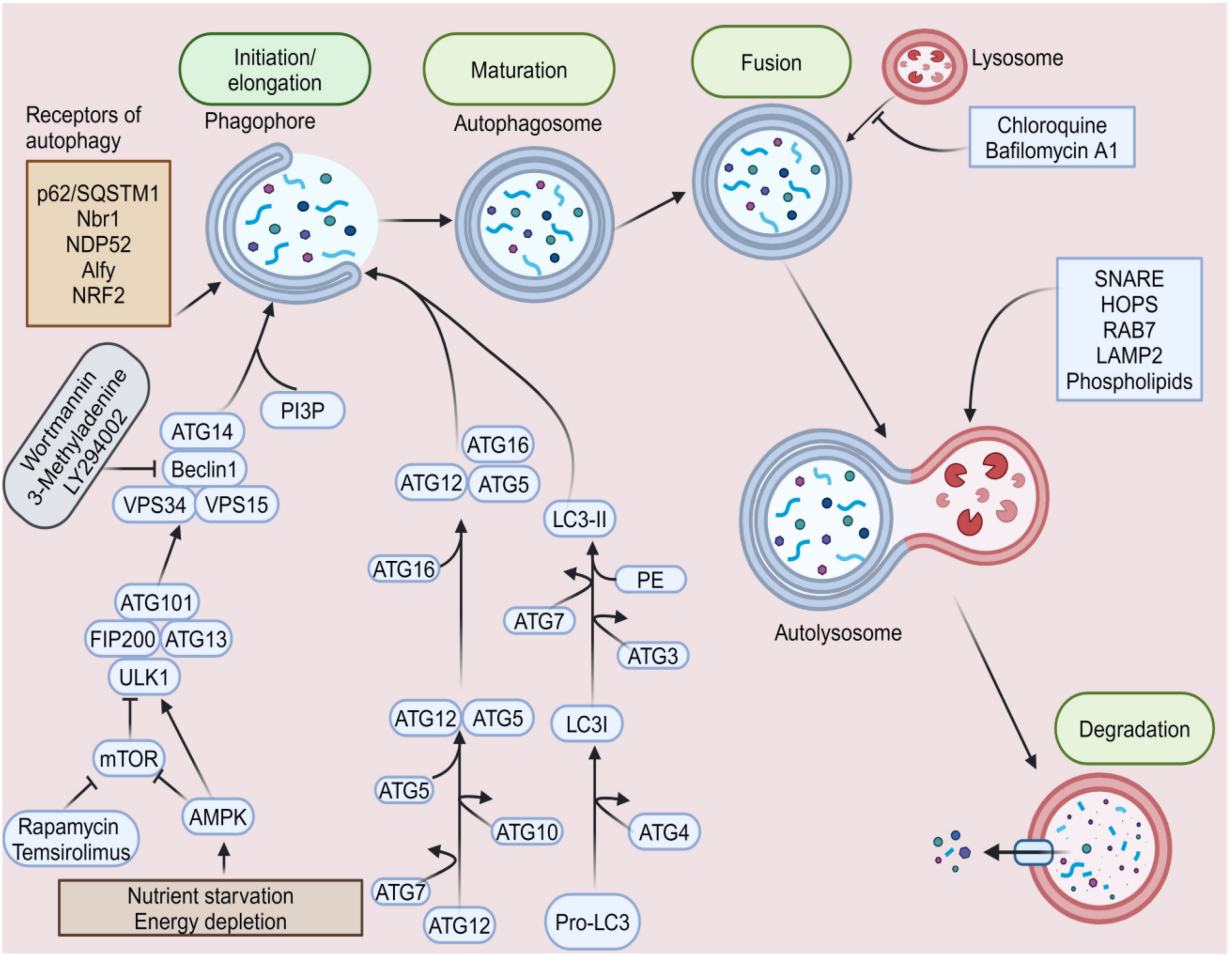
Autophagy pathway. Nutrient starvation and energy depletion cause several cascades that alter the phagophore’s initiation and elongation to the autophagosome. During this process, ubiquitin-like conjugation cascade systems such as the LC3s and ATGs facilitate autophagosome formation. AMPK, amp-activated protein kinase; ATG, autophagy-related gene; FIP200, focal adhesion kinase family interacting protein of 200 kDa; HOPS, homotypic fusion and protein sorting; LAMP-2, lysosome-associated membrane protein 2; LC3, microtubule-associated protein 1 light chain 3; mTOR: mammalian target of rapamycin; Nrf2, nuclear factor erythroid 2-related factor 2; PI3P, phosphatidylinositol 3-phosphate; SNARE, soluble N-ethylmaleimide-sensitive factor attachment protein receptor; ULK1, unc-51-like kinase 1; VPS 34, vacuolar protein sorting 34.

A popular marker for macroautophagy tests is LC3. Following translation, ATG4 processes pro-LC3 to reveal the glycine residue at the C-terminus to facilitate downstream conjugation events that convert LC3-I to LC3-II (137). Once autophagosomes fuse with lysosomes, autophagy consumes biological components in double membrane-bound autophagosomes for recycling and clearance (138). Transcription factor EB (TFEB) enhances autophagic flow by promoting lysosome formation, generating autophagosomes, and fusing with lysosomes, thereby aiding in the clearance of harmful protein structures (139). Soluble N-ethylmaleimide-sensitive factor attachment protein receptors (SNAREs), RABs, and tethering complexes (homotypic fusion and protein sorting (HOPS)-tethering complex) are mainly responsible for controlling membrane fusion (140). **Figure 4** illustrates the autophagy pathway.

### 5.1 Autophagy in IBD

#### 5.1.1 Autophagy genetic polymorphisms and risk of IBD

Multiple autophagy gene variants have been linked to an elevated risk of IBD. An elevated risk of CD is associated with the AG genotype for rs2241880 (*ATG16L1*) in Iraqi patients (141). Similarly, *ATG16L1* rs2241880 (G allele*)* is a consistent risk factor for CD in Caucasian populations, according to a meta-analysis (142). In a different study, the T and G alleles of *ATG16L1* rs2241880 polymorphisms are associated with an increased risk of CRC (143) and esophageal cancer (144), respectively.

In Indian patients, the T allele at rs4663402 (*ATG16L1)* and the C allele at rs4663421 (*ATG16L1*) are positively associated with CD and UC (145). Among Iranians, there is a noteworthy correlation between the *ATG16L1* gene rs2241879 and an elevated risk of IBD (146). These results show that genetic variations in the *ATG16L1* may lead to an increased risk of IBD.

#### 5.1.2 Expression levels of autophagy genes/proteins in IBD

Rezaie and colleagues discovered the downregulation of autophagy-related genes in the colon of the DSS group, including *Beclin*, *ATG12*, *ATG5*, *ATG7*, and *ATG13* (147). Another study found that the colitis animals exhibit significantly increased autophagy-related proteins like mTOR, P62, and p-MTOR in IHC but substantially reduced LC3B levels (148). In western blot analysis, a similar pattern is seen. In the colon tissue of mice with DSS-induced colitis, the expressions of P62, mTOR, and p-mTOR increased, while ATG16L1 and LC3II/I decreased (148). Another study also showed that the DSS group exhibits an increase in p62 expression, while a decrease in the LC3II/I ratio and Beclin-1 expression is observed (149). Through western blot analysis, Shi and his team found increased levels of p62, p-mTOR/mTOR, and LC3-II/LC3-I in DSS-treated mice but decreased levels of Beclin-1 (150).

mTOR silencing significantly reduced inflammation and oxidative damage caused by LPS, but blocking ATG5 increased these effects. Experimental colitis and oxidative stress were significantly reduced *in vivo* by the pharmacological injection of mTOR inhibitors and autophagy stimulators (151). This further provides evidence that mTOR may contribute to IBD pathogenesis.

#### 5.1.3 Autophagy regulators in clinical studies

Activating transcription factor 4 (ATF4) controls genes related to ER stress, autophagy, amino acid metabolism, and the inflammatory response. In patients with active CD or UC, the inflammatory intestinal mucosa has lower levels of ATF4. ATF4 loss in mice decreases *Slc1a5* transcription, which decreases glutamine absorption by IECs and antimicrobial peptide expression. Therefore, ATF4 may be a target for IBD treatment (152).

CD-associated mutations alter the autophagy-mediated antibacterial pathway involving ATG16L1 and NOD2 in a manner specific to certain cells or functions (153). Studies have shown that NDO2 polymorphisms lead to IBD susceptibility. For instance, Watson et al. (154) found that patients with very early-onset IBD who have *NOD2* polymorphisms *(NOD2+)* were substantially more likely than those in the *NOD2* group to have arthropathy (60%) and a CD-like phenotype (90%), as well as linear growth impairment (90%).Horowitz et al. (155) found that a molecular driver of early onset IBD, specifically CD, is the recessive inheritance of *NOD2* alleles, most likely a consequence of impaired NOD2 protein activity. Abdelnaby and the team also found that among Kuwaiti CD patients, *NOD2/CARD15* gene variants were substantially linked to an elevated risk of illness and aggressive characteristics (156).

Human immunity-related GTP-binding protein M (IRGM) regulates mitophagy and xenophagy, two forms of selective autophagy (157). Lu and colleagues found that polymorphisms in the autophagy gene IRGM seem to increase the risk of CD but not UC, particularly among Europeans. This could help clarify the part autophagy plays in the pathophysiology of CD (158). IRGM participates in autophagy and mediates innate immune responses (159). It has been shown that the *IRGM* gene’s single-nucleotide polymorphism rs4958847 showed a highly significant correlation with the incidence of surgery in ileocolonic CD patients (159).

#### 5.1.4 Polymorphisms in autophagy and gut microbiota

The risk allele *ATG16L1* T300A, a single nucleotide polymorphism (SNP) linked to increased CD risk, leads to dysbiosis in mice, causing an increase in Bacteroides and amplifying the Th1 and Th17 immune responses in the gut lamina propria (160). These alterations occur before the start of illness in human stool microbiome-associated mice, indicating that microbiota modifications cause inflammatory cell population shifts in the gut (160). These findings clarify the genesis of CD and shed light on the connection between SNPs, dysbiosis, and the gut’s immune system (160). Another study revealed that ATG16L1^T300A/T300A^ mice display several bacteria linked to IBD, including *Tyzzerella, Mucispirillum, Ruminococcaceae*, and *Cyanobacteria*. In the DSS colitis paradigm, ATG16L1^T300A/T300A^ mice exhibited more severe inflammation than wild-type mice (161).

#### 5.1.5 Autophagy and immune cells/immune response

Zhang and team found that *ATG16L1* deficiency in dendritic cell (DC) mice displays elevated pro-inflammatory TNF-α and IL-1β levels, leading to intestinal inflammation. Thus, one of the unique causes of IBD is decreased ATG16L1 activity, resulting in elevated pro-inflammatory cytokines *in vivo* (162). Similarly, the deletion of *ATGI6L1* in CD11c+ DCs exacerbates intestinal inflammation in DSS-induced colitis. The deletion of *ATG16L1* enhances the co-expression of RAB5 and RAB7 with *Salmonella typhimurium* but doesn’t affect Beclin1 and suppresses the co-expression of LC3 and LAMP1 (163). The study indicates that *ATGI6L1* deletion in the presence of *Salmonella typhimurium* can exacerbate colitis. This may suggest that *ATGI6L1*’s presence protects against colitis aggravation while its variants lead to IBD. As a result, *ATGI6L1* and its variants may play distinct roles in immune system regulation during IBD progression.

Plantinga and team found that the *ATG16L1* polymorphism in humans is linked to elevated IL-1β and IL-6 production, potentially influencing the inflammatory process in CD. Cells from *ATG16L1* Thr300Ala (T300A) risk variants shown to affect ATG16L1 protein expression show enhanced NOD2-stimulated production of the pro-inflammatory cytokines IL-1β and IL-6 (164). In a different study, the *ATG16L1* T300A is linked to better survival in gastric cancer individuals (165). The study found that tumors of individuals with T300A/T300A have downregulated PPAR, EGFR, and inflammatory chemokine pathways, while Wnt/β-catenin signaling is upregulated (165). This implies that the *ATG16L1* T300A may have different roles in IBD and gastric cancer.

## 6 Ferroptosis

Ferroptosis is a controlled cell death influenced by iron and severe lipid peroxidation (LPO), affecting various physiological and pathological processes (166). An important component of ferroptosis is the transferrin receptor, which is essential for intracellular iron buildup and the development of ferroptosis (167). Iron is typically transported to endosomes by transferrin receptors, where the six-transmembrane epithelial antigen of prostate family member 3 (STEAP3) converts it from Fe3+ to Fe2+ (168). The plasmalemma divalent metal ion transporter 1 (DMT1) facilitates the cellular uptake of Fe2+, while transferrin receptors carry transferrin-bound Fe3+ (169). Through poly r(C)-binding protein 1 (PCBP1), the labile iron pool (LIP) is coordinated, allowing the cell to effectively transport iron to non-heme iron enzymes, store iron in ferritin, and provide iron for the Fe-S cluster assembly/repair mechanism (170). Ferroportin (FPN), the only known iron exporter, is crucial for maintaining iron homeostasis (171). Ferritin is transported to autophagolysosomes for breakdown by nuclear receptor coactivator 4 during ferritinophagy (172). After autophagy degrades ferritin, iron ions are released, which trigger the LIP to initiate the fenton reaction, leading to lipid peroxidation (172). A study reveals that Z-Ligustilide’s excessive activation of the nuclear factor erythroid 2-related factor 2 (Nrf2)/heme oxygenase-1 (HO-1) pathway is responsible for the selective onset of ferroptosis in leukemia cells. The primary cause is the ROS-induced accumulation of LIP in acute myeloid leukemia cells (173).

An intracellular antioxidant called glutathione (GSH) is produced from glutamate, cysteine, and glycine (174). Free cystine enters cells via the cystine-glutamate antiporter xCT, while plasma glutathione-disulphide may be the main source of cystine throughout the body (175). A study in acute myeloid cells found that reduced cystine and glutamine levels disrupt GSH synthesis, leading to the malfunction of glutathione peroxidase-4 (GPX4), a co-factor used to maintain lipid peroxidation homeostasis (176). Small compounds that inhibit GPX4 generate a fatal buildup of lipid peroxides and promote ferroptosis cell death (177). A study by Cheng and the team found that Leonurine raises GPX4 and GSH, fixes ultrastructural defects in mitochondria effectively, and greatly lowers ferroptosis in acute kidney injury (AKI), both *in vivo* and *in vitro*. It also considerably reduces endoplasmic reticulum (ER) stress by downregulating activating transcription factor 4 (ATF4), CHOP, and Chac glutathione-specific γ−glutamylcyclotransferase 1 (CHAC1) (178). This may imply that in ER stress, the upregulation of ATF4, CHOP, and CHAC1 may suppress GSH and GPX4, leading to ferroptosis. Another study found that the GSH/glutathione disulphide ratio decreases when cells are exposed to dihydroartemisinin (DHA), a ferroptosis inducer. Treatment with DHA also inhibits GPX4 and increases CHAC1 expression levels (179). Lipid peroxidation, which may result from GPX4 activity suppression, can cause ferroptosis (180).

The long-chain acyl-coenzyme A synthase 4 (ACSL4) esterifies coenzyme A (CoA) to produce certain polyunsaturated fatty acids (PUFAs), including adrenic and arachidonic acid. The production of arachidonoyl-CoA, facilitated by ACSL4, plays a crucial role in ferroptosis execution by promoting phospholipid peroxidation (181). TPCI (photosensitizer) produces ROS when exposed to light, activating ALOX12 or resuscitating it through SLC7A11 downregulation. This leads to direct peroxidation of PUFAs into fatal lipid ROS, causing ferroptosis in cancer cells independent of ACSL4 (182). The study suggests lipid peroxidation may occur through arachidonate 12-lipoxygenase (ALOX12) activation when solute carrier family (SLC) 7A11 is down-regulated without ACSL4.

Recent research has revealed a connection between ferroptosis and p53 (183). Ren and colleagues found that the p53/spermidine/spermine N1-acetyltransferase 1 (SAT1)/arachidonic acid 15-lipoxygenase (ALOX15) signaling pathway induces ferroptosis, which is significantly suppressed by cerebroprotein hydrolysate-I in Alzheimer’s disease (184). The expression of SAT1 causes LPO and makes cells more susceptible to ferroptosis in response to stress caused by ROS. In xenograft tumor models, this results in tumor growth inhibition (185). However, in xenograft mouse models, inhibiting endogenous independent phospholipase A2β (iPLA2β) causes tumor cells to undergo p53-driven ferroptosis, increasing p53-dependent tumor suppression (186). This implies that the activation of iPLA2β may prevent p53-driven ferroptosis.

**Figure 5:**
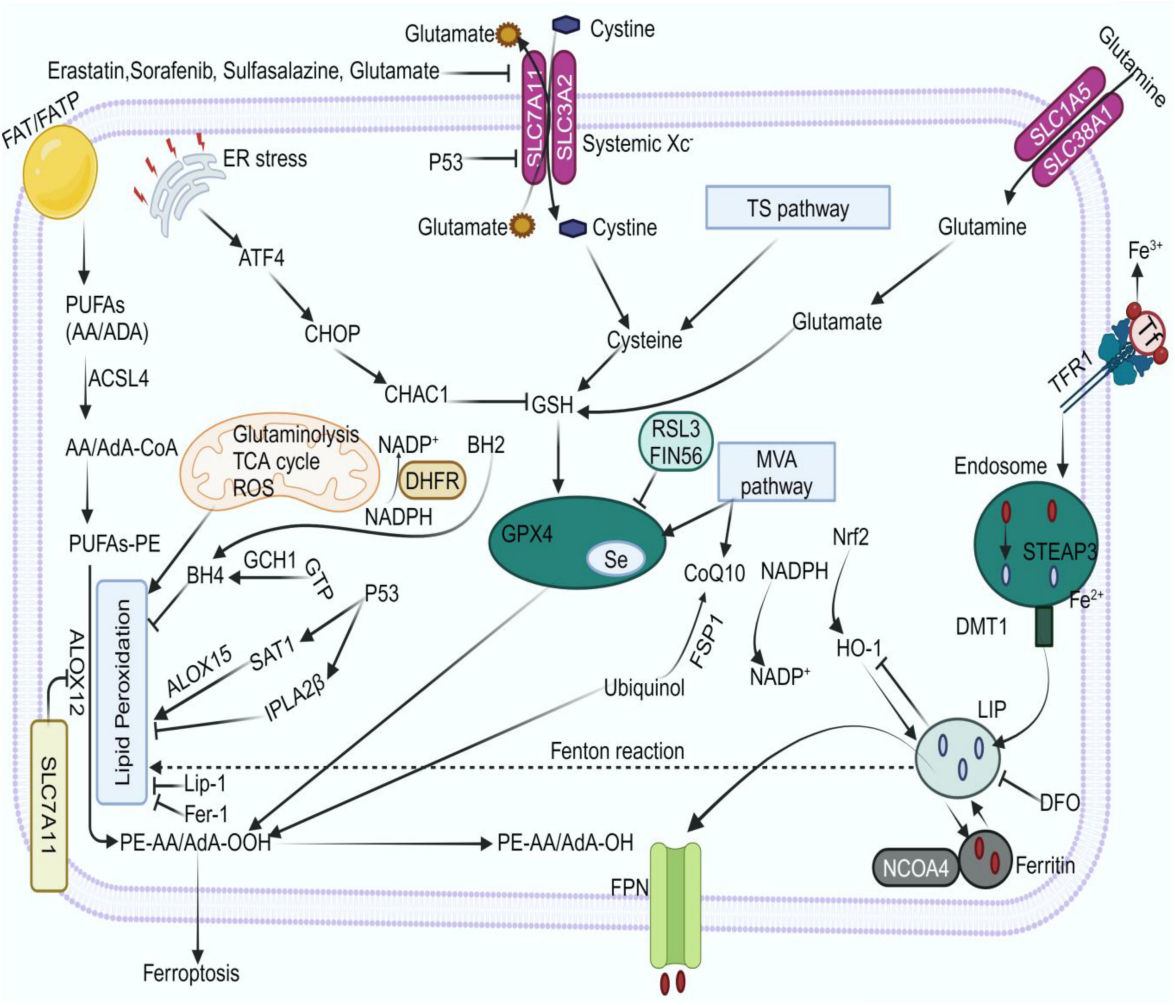
Mechanism of ferroptosis. TFRI/TF, FAT/FATP/PUFAs, and other pathways influence ferroptosis formation. Lip-1, DFO, Fer-1, the GCH1/BH4 and GSH/GPX4 pathways, and FSP1 suppress ferroptosis. ACSL4, long-chain acyl-coenzyme A synthase 4; ALOX12/5, arachidonate 12/5-lipoxygenase; ATF4, activating transcription factor 4; BH2, dihydrobiopterin; BH4, tetrahydrobiopterin; CHACI: chac glutathione specific γ−glutamylcyclotransferase 1; DFO, deferoxamine; DHFR, dihydrofolate reductase; DMT1, divalent metal ion transporter 1; ER, endoplasmic reticulum; Fer-1, ferrostatin-1; FPN, ferroportin; FSP1, ferroptosis suppressor protein 1; GCH1, cyclohydrolase-1; GPX4, glutathione peroxidase-4; GSH, glutathione; GTP, guanosine 5′-triphosphate; HO-1, heme oxygenase-1; iPLA2β, independent phospholipase A2β; LIP, labile iron pool; Lip-1, liproxstatin-1; MVA, Mevalonate; NADP^+^,nicotinamide adenine dinucleotide phosphate; NADPH, nicotinamide adenine dinucleotide phosphate; NCOA4, nuclear receptor coactivator 4; Nrf2, nuclear factor erythroid 2-related factor 2; PUFAs, polyunsaturated fatty acids; ROS, reactive oxygen species; SATI, spermine N1-acetyltransferase 1; SLC, solute carrier family; STEAP3, six-transmembrane epithelial antigen of prostate family member 3; TCA, tricarboxylic acid; TFR1, transferrin receptor 1; TS, transsulfuration.

Certain elements have been found to trigger lipid peroxidation, leading to ferroptosis. Like erastin and RSL3, which block system XC- or directly target the reducing enzyme GPX4, respectively, FINO2 does not deplete GPX4 protein, unlike FIN56. Rather, FINO2 directly oxidizes iron and indirectly inhibits GPX4’s enzymatic activity, leading to widespread lipid peroxidation (187). However, GTP cyclohydrolase-1 (GCH1)-expressing cells synthesize tetrahydrobiopterin (BH4)/dihydrobiopterin (BH2), which results in lipid remodeling and inhibits ferroptosis by blocking phospholipid loss with two acyl tails of polyunsaturated fats (188). BH4 is a strong antioxidant that sequesters radicals and, either by itself or in combination with vitamin E, prevents lipid membranes from undergoing autoxidation (189).The transsulfuration pathway, mevalonate pathway, ferroptosis inhibitory protein 1 (FSP1)-coenzyme Q10 (CoQ10) pathway, dihydroorotate dehydrogenase (DHODH)-dihydroubiquione (CoQH2) pathway, and GTP cyclohydrolase-1 (GCH1)-tetrahydrobiopterin (BH4) pathway are among the other antioxidant systems that have also been linked to the regulation of ferroptosis (190) (**Figure 5**).

### 6.1 Ferroptosis in IBD

#### 6.1.1 Ferroptosis in IEC

The mammalian GIT, containing innate and adaptive immune cells and trillions of commensal microbes, requires barrier and regulatory systems to maintain tissue homeostasis and host-microbial relationships (191). Therefore, IECs are crucial mediators in maintaining intestinal homeostasis, promoting the formation of an immune environment conducive to commensal bacterial colonization (191). Notably, it has been shown that ferroptosis disrupts the IECs, which causes IBD. Xu and team found that IECs from UC patients and colitis-affected rats exhibit markedly increased ferroptosis, driven by ER stress signaling (192). Another study by Chen and colleagues also found that SLC6A14 uses the C/EBPβ-PAK6 axis to help epithelial cells undergo ferroptosis in UC (193). Mucosal inflammation, characterized by an impaired intestinal epithelial barrier, exposes the immune system to more luminal bacteria, leading to an ongoing inflammatory response (194).

According to *in vitro* studies, ACSL4 plays a significant role in the IEC impairment brought on by LPS stimulation. Using si-ACSL4 or RSG to inhibit ACSL4 can provide efficient defense against intestinal epithelial damage brought on by LPS (195). In Caco2 cells, ACSL4 siRNA significantly reduced the hypoxia-induced production of ACSL4, elevated the expression of GPx4, and reduced lipid peroxidation. By blocking ischemia-induced ACSL4, intestinal ischemia/reperfusion-induced cell damage and intestinal barrier dysfunction were lessened, and ferroptosis and lipid peroxidation were prevented (196). These findings suggest that ferroptosis can lead to IEC damage/disruption.

#### 6.1.2 Ferroptosis in immune cells

##### 6.1.2.1 Regulatory T cells

Tregs are crucial for sustaining immunological tolerance and homeostasis by regulating immune system activation (197). Treg cells are essential to the complex pathophysiology of IBD at the beginning or development of the disease (198). Preclinically, studies have shown that Tregs may undergo ferroptosis. Yan and their team found that a high-fat diet causes intestine Treg cells to undergo ferroptosis, which may be the primary first step in immunotolerance loss and colitis development (199). The reduction of Treg cells in necrotizing enterocolitis (NEC) is ascribed to ferroptosis caused by decreased expression of GPX4. Treg cells with GPX4 deficiency have reduced immunosuppressive activity and are prone to ferroptosis. In NEC, the ferroptosis of Treg cells worsens damage to the gut and increases the inflammatory cell response (200).According to Xu and the team, GPX4 controls immunological homeostasis and antitumor immunity by preventing Treg cells from ferroptosis and lipid peroxidation. GPX4 loss causes excessive lipid peroxide buildup and Treg cell ferroptosis in response to T cell receptor (TCR)/CD28 co-stimulation (201). These imply that Tregs may undergo ferroptosis.

##### 6.1.2.2 Macrophages

As antigen-presenting cells, macrophages release molecules that modulate the immune system, such as chemokines and cytokines, essential for triggering other intestinal immune cells and influencing the gut’s immunological response (202). Macrophages, responsible for innate immunity, also play a role in the development of intestinal inflammation (203). Recent research indicates that ferroptosis in macrophages can lead to the development of colitis. Ye and colleagues found that combining ferrostatin-1 (Fer-1) and 5-aminosalicylic acid reduces ferroptosis in colon tissue macrophages and increases M2 macrophages, suggesting targeting ferroptosis in M2 macrophages as a potential treatment for UC. This shows that macrophages may undergo ferroptosis. The study further demonstrated that M2 macrophages are more susceptible to ferroptosis than M1 macrophages, and this vulnerability is linked to the ERK-cPLA2-ACSL4-mediated activated arachidonic acid (AA) metabolism pathway (204). Another study also showed that mice with UC exhibit increased Fe2 accumulation in their colon macrophages, linked to increased production of inflammatory cytokines like NO, IL-1β, TNF-α, and IL-6 (205). Fe2+ accumulation is known to cause ferroptosis; therefore, increased Fe2 in macrophages may lead to macrophage ferroptosis. In a different study, ferroptotic macrophages facilitate the inflammatory bone resorption linked to apical periodontitis (206).

##### 6.1.2.3 Group 3 innate lymphoid cells

ILC3 and intestinal T cells regulate gut immune responses and the microbiota’s makeup (207). ILC3s are vital for maintaining intestinal tissue integrity and defending against pathogens, and their dysfunction can increase vulnerability to gut inflammation. Intestinal mucosal ILC3s from patients with UC are shown to have elevated levels of ferroptosis-related genes, including GPX4, a crucial ferroptosis regulator (208). In a mouse model, when GPX4 was deleted, the number of NKp46+ILC3 cells decreased, IL-22 and IL-17A production was compromised, and intestinal inflammation worsened in a way that was independent of T cells (208). These findings suggest that ILC3 may undergo ferroptosis.

#### 6.1.3 Ferroptosis-related genes as biomarkers

Biomarkers are utilized in various fields, such as diagnosing IBD, assessing disease activity, predicting treatment impact, and predicting relapse (209). Therefore, it is necessary to have biomarkers that combine environmental and genetic elements to forecast the course of complicated immunological illnesses like IBD (210). Certain genes involved in ferroptosis in IBD have been discovered. These genes may have diagnostic value for IBD. A study indicates that UC is linked to STAT3-mediated ferroptosis, suggesting that STAT3, a gene linked to ferroptosis, could serve as a valuable biomarker for diagnosis and treatment (211). Another study by Qian et al. (212) identified five hub genes *(LCN2, MUC1, PARP8, PLIN2*, and *TIMP1*) that can differentiate UC patients from controls and positively associate with ferroptosis in UC. These genes positively correlate with M1 macrophages and neutrophils. The logistic approach had an AUC value of 1.000 for the training cohort and 0.995 for the validation cohort. Therefore, these hub genes may be useful in diagnosing UC from controls. Similarly, five ferroptosis-related hub genes such as *ALOX5, TIMP1, TNFAIP3, SOCS1*, and *DUOX2* have been identified as diagnostic markers to differentiate between UC and controls. *SOCS1, TIMP1, DUOX2, and ALOX5* negatively correlate with M2 macrophages, while *ALOX5* and *TNFAIP3* are positively connected with neutrophils (213).

Other ferroptosis-related genes have been identified in CD. Ji and the team found five ferroptosis-related hub genes such as *PTGS2, IL6, IL1B, NOS2*, and *IDO1*. The expression of hub genes in CD patients and normal subjects showed significant changes upon external validation (214). The AUC values for all genes were above 0.8, suggesting they could serve as CD biomarkers (214). Zhang et al. (215) also discovered three upregulated ferroptosis genes (*IL-6, DUOX2*, and *PTGS2)* likely to modulate ferroptosis in CD and may be involved in its development and progression. Therefore, the findings could lead to new CD biomarkers and diagnostic and therapeutic indicators.

#### 6.1.4 Gut microbiome and ferroptosis

Recently, the pathophysiology of IBD has been linked to the adherent-invasive *Escherichia coli* (AIEC) pathotype of *E. coli (* 216). Therefore, a recent study has shown that AIEC causes ferroptosis in IECs. AIE Ccolonisation in CD patients’ terminal ileum increases 4-hydroxynonenal levels and decreases ferritin heavy chain (FTH) and GPX4 levels in the intestinal epithelium (217). *In vitro* tests show that AIEC infection can lower FTH and GPX4 levels, elevate LPO, and cause IEC ferroptosis (217). So AIEC may modulate GPX4, FTH, and LPO to trigger ferroptosis.

## 7 Cell death in IBD pathophysiology

IEC passive shedding largely involves apoptosis at villi tips (218). In mice, shed IECs have been shown to persist for several hours, promoting the expression of antimicrobial genes at the tips of villi and helping to control the makeup of the gut microbiota (219).The rate of senescent epithelial monolayer cell clearance and the growth of stem cells in the crypts are both correlated with the shedding and renewal of IECs (21). It is unclear exactly how IEC death occurs in IBD (21). However, IEC shedding is elevated, and barrier integrity is compromised in the intestinal lamina propria due to a highly inflammatory environment rich in the proinflammatory cytokine TNF-α, which further fuels inflammation (21). Also, in IBD, IECs are continuously lost, and other immune cells are also continuously shed; this is most noticeable during the active stages of the disease. In IBD, the digestive tract experiences excessive cell death as a result of ongoing inflammation and recurring bouts. Increased cell death may stimulate the gut immune system, exacerbating intestinal inflammation in IBD (220, 221). Excessive IEC apoptotic cell death during the pathophysiological state causes a chronic inflammatory condition (222). Later, the necroptotic cell death takes over, bringing about more pathological features than apoptosis (222). It may also trigger other lytic cell death mechanisms, such as ferroptosis and pyroptosis, to increase the pathogenesis of intestinal diseases (222).These findings suggest that excessive cell death may destabilize the barrier and activate immune cells, resulting in additional inflammation. The commencement of apoptotic cell death may trigger the activation of other cell death mechanisms such as necroptosis, ferroptosis, and pyroptosis. As a result, cell death in IBD may activate other cell death processes, and in the presence of an inflammatory environment, the vicious cycle of cell death persists.

IBD-related necroptosis mostly affects IECs, and RIPK3 inhibition can somewhat reduce the chronic intestinal inflammation brought on by necroptosis (223, 224). In IBD, intestinal stem cells may also undergo necroptosis. The loss of the essential gene *SETDB1* can cause necroptosis, which alters colon epithelial differentiation, compromises the mucosal layer, and increases intestinal inflammation (225, 226). According to a study on IBD patients, RIPK3-induced necroptosis modifies occludin, zonulin-1, and E-cadherin, which impacts membrane permeability (120). In the IBD gut, cell pyroptosis is caused by inflammasome activation (e.g., NLRP3), essential for innate immune reactions, and is vital for gut-brain balance and gut microbiota maintenance (227, 228). Cell pyroptosis, which is mostly carried out by proteins like GSDMB, GADMD, and GSDME, mediates several damage signals that result in chronic inflammation that persists in IBD (229). Additionally, GSDMB, a pyroptosis executor, is essential for preserving the function of the epithelial layer and reducing inflammation in IBD (230). Necroptosis and pyroptosis can cause lytic cell death, which is probably why they have the potential to spread disease. Distinct from apoptosis, this type of cell suicide permits the release of immunogenic cellular material, such as inflammatory cytokines like interleukin-1β (IL-1β) and damage-associated molecular patterns (DAMPs), to cause inflammation (231). Ferroptosis is seen in IECs of DSS animals and IBD patients, mostly due to endoplasmic reticulum stress and the NF-κB pathway (232). In IBD, the intestinal epithelium experiences excessive lipid peroxidation, elevated ferrous iron levels, and ROS buildup, contributing to chronic aberrant inflammation (232, 233). Ferroptosis inhibitors have demonstrated efficacious management of intestinal chronic inflammation, a finding extensively confirmed in both animal model and IBD patients (211, 234, 235).

Intestinal homeostasis and repair depend on autophagy and its regulatory mechanisms, promoting intestinal barrier function in response to cellular stress by regulating tight junctions and preventing cell death. Moreover, it has become evident that autophagy plays a part in intestinal stem cells as well as secretory cells, influencing their metabolism as well as their ability to proliferate and regenerate (236).

In addition, TNF-α can cause RIPK3-dependent necroptosis and extrinsic caspase-8 and executioner caspase-3-dependent apoptosis when caspase-8 or TNFAIP3 (A20, a ubiquitin editing enzyme) capabilities are compromised (223, 237–241). TNF-α can also cause IEC shedding (242–245). Unlike homeostatic IEC shedding where barrier integrity is maintained by rapid basolateral tight-junction protein redistribution and zipper-like replacement by neighboring cells (246, 247), necroptosis, in which several nearby IECs lose contact, has been documented to accompany TNF-α-induced shedding (243, 248). It would be challenging to discern cause from effect if greater IEC death and barrier integrity resulted in more inflammation, intestinal epithelial damage, and possibly even dysbiosis (21). Additional elements that might cause cell death include oxidative stress, hypoxia, and endoplasmic reticulum (ER) stress (249). Cell death can result in aberrant IECs, which attract immune cells and promote inflammation. At the same time, inflammatory cytokines like TNF can trigger cell death, which leads to IEC abnormalities. As a result, cell death is bidirectional, making it difficult to discern the causal link between cell death and inflammation.

As previously documented, IEC cell death leads to IEC irregularity, which attracts immune cells and promotes inflammation. Meanwhile, inflammatory cytokines such as TNF-α can cause cell death, resulting in IEC abnormality. Therefore, cell death is a bidirectional process, and it may be difficult to determine the causal relationship between cell death and inflammation. Therapies targeting the upstream cytokines such as TNF-α and IL-1β may be the best option for treating cell death, which can help reduce cell death at the IEC or immune cells. This may prevent proapoptotic signals. It is also known that excessive IEC apoptotic cell death leads to chronic inflammation, followed by necroptotic cell death. This causes more pathological features and potentially triggers other lytic cell death mechanisms like ferroptosis and pyroptosis, increasing the pathogenesis of intestinal diseases. Therefore, preventing the upstream signal TNF-α may prevent cell death. Some patients may not react to anti-TNF therapy or experience diminishing response over time. Targeting cell death markers with MSCs may prevent cell death, gut abnormalities, and inflammation, which may prevent further cell death processes. Therefore, exploring combination therapy with IBD medications and MSCs is recommended for better therapeutic outcomes.

## 8 MSCs and cell death modulation in IBD

MSCs are a crucial alternative for tissue healing due to their differentiation capacity and paracrine characteristics. MSCs release extracellular vesicles (exosomes and microvesicles) and secrete soluble substances, fulfilling their paracrine roles. Extracellular vesicles, primarily endosomal in origin, carry proteins, mRNA, and miRNA from the cells of origin to target cells. Recent research indicates that MSCs’ therapeutic impact in animal disease models is solely due to these extracellular vesicles, suggesting they could replace MSC-based therapy in regenerative medicine (250). Nearly every tissue contains MSCs, which develop into specific cell types and perform immunomodulatory actions (251). MSCs primarily engage in immunomodulatory activities through cell-to-cell interactions with immune cells, including T cells, B cells, natural killer (NK) cells, macrophages, monocytes, dendritic cells (DCs), and neutrophils (252). Therefore, MSCs may regulate cell death through their extracellular vesicles (EVs) and interactions with immune cells. Major cell death markers in IBD lead to greater epithelial cell loss, decreased intestinal barrier integrity, and increased inflammatory cytokines and alarmins. DSS, 2,4,6-trinitrobenzene sulfonic acid (TNBS), and LPS have been utilized to create cell death models in tissues and cells. MSCs’ ability to regulate these markers, prevent epithelial cell loss, improve intestinal barrier integrity, and reduce inflammation may help prevent IBD. These will promote cell survival, tissue regeneration, immune cell modulation, and reduce inflammation.

### 8.1 MSC and apoptosis

Nishikawa and the team discovered that the DSS-induced colitis mouse model showed higher concentrations of caspase-3-positive cells and apoptotic nuclear cells in the colon sections and increased colon inflammatory cytokines, leading to decreased intestinal tight junction proteins such as claudin-2 and occludin (253). However, constant filtrated murine adipose-derived MSC lysate (FADSTL) administration prevented apoptosis, reduced inflammation, leading to preserved tight junction proteins (increased claudin-2 and occludin), and alleviated clinical symptoms (253). Yang et al. (254) found that rats that received TNBS enema experienced severe diarrhea, mucosal injury, weight and appetite loss, decreased colon length, elevated inflammatory markers, and even bloody stools. Additionally, there was an increase in the cleavage of apoptotic markers such as caspase-3, caspase-8, and caspase-9 (254). However, BMMSC-EVs reduced and averted all the colon pathologies and inhibited apoptosis in colitis rats by decreasing caspase-3, caspase-8, and caspase-9 cleavage (254). Liu and the team discovered that BMMSC-conditioned medium (CM) reduces cell apoptosis in DSS-induced experimental colitis. Proinflammatory cytokines, a shorter colon, weight loss, bloody diarrhea, lower expression of ZO-1, anti-apoptotic protein Bcl-2, and greater abundance of the pro-apoptotic proteins Bax, caspase 3, and cleaved caspase 3 were all seen in the DSS group. BMMSC-CM enema therapy increased ZO-1, prevented cell apoptosis, and reduced all histopathological characteristics (255). Additionally, MSC-CM decreased macrophage and neutrophil recruitment while augmenting the concentration of Foxp3 + Tregs (255). In a different study, the Bax/Bcl-2 ratio was higher in mice with colitis-associated cancer. However, treatment with intestinal MSCs reduced this ratio, providing protection against colitis-associated cancer and improving colitis symptoms (256). Xu and the team found that the infusion of human embryonic stem cells (T-MSCs) into mice reduced colitis by increasing the level of IGF-1 in the blood. Epithelium loss and inflammatory cell infiltration were increased, but T-MSC reversed these changes. The study found that 50 ng/mL TNF-α caused apoptosis in the human colon epithelial cell line (NCM 460 cells) *in vitro*, but rhIGF-1 stimulation reduced the proportion of early apoptotic cells, as per Annexin V and 7-AAD staining flow cytometry. Higher levels of IGF-1 helped to repair and regenerate epithelial cells while preserving their integrity. IGF-1-treated organoids were found to be larger and to have more buddings in an *in vivo* investigation that replicated the *in vitro* results. Additionally, on day 10, the number of organoids detected increased (257). It has been demonstrated that IGF-1 protects against apoptosis generated by intrinsic pathways but not by extrinsic pathways (258). Therefore, it is possible that T-MSC may have reduced apoptosis of epithelial cells, hence preventing colitis *in vivo*. Yousefi-Ahmadipour et al. (259) found that in the colitis rats, the expression level of the antiapoptotic protein Bcl-2 was dramatically reduced, whereas that of the proapoptotic protein Bax was significantly elevated. Additionally, rats given TNBS experienced severe bloody diarrhea, an increased colon weight-to-length ratio, a macroscopic damage score, goblet cell loss, submucosal edema, increased inflammatory cell infiltration, and a marked weight loss (259). Nonetheless, concurrent administration of ASCs and sulfasalazine reversed all these changes. The combination also converted inflammatory M1 macrophages into anti-inflammatory M2 macrophages by increasing IL-10 and Arg-1 levels, decreasing MCP1 and CXCL9 levels, and promoting T reg cell development via Foxp3 gene activation (259). This shows the role of combination therapy with MSC and IBD medications. In a different study, adipose-derived MSCs reduce alveolar hemorrhage, thickening of the alveolar walls, and inflammatory infiltration caused by radiation in mice’s lung tissue. The MSCs increased lung tissue cell regeneration and decreased radiation-induced cell apoptosis (260). Yang and the team revealed that the number of apoptotic cardiomyocytes dropped in the BMMSCs exosome and BMMSCsDSY exosome groups. Cleaved caspase-3 expression levels decreased in the BMMSC and BMMSCsDSY exosome groups, with the latter group exhibiting lower expression levels (261). On days 7 and 28, the BMMSCs exosome group experienced an increase in the BAX/BCL2 ratio, while the BMMSCsDSY exosome group experienced a decrease (261).

Sun et al. (262) found that cell activity dropped, and early apoptosis happened following IEC-6 treatment with TNF-α and lymphocytes. ZO-1 concentrations dropped. The study found that treatment with HO-1/bone marrow (BM) MSC-derived exosomes significantly improves cell status and IEC-6 survival, reduces early apoptotic cells, and protects tight junction structures, demonstrated by increased ZO-1in an inflammatory environment. The HO-1/BMMSCs-exosomes group displayed a lower proportion of cleaved caspase-3 and BAX/BCL2 ratio compared to the BMMSCs co-culture group (262). Also, MSCs-EVs (from BM) release miR-378a-3p, which blocks GATA-binding protein 2 (GATA2), downregulating aquaporin-4 (AQP4) expression, and disrupting the peroxisome proliferator-activated receptor-α (PPAR-α) signaling pathway. This prevents the formation of IBD by suppressing the LPS-induced apoptosis of M064 cells (263). Results from an LPS-induced human colonic epithelial cell model show that the LPS group had higher levels of caspase-3 and Bax expression than the control group. While claudin-1 and ZO-1 expression declined in the LPS group, relative TNF-α expression rose (264). Nevertheless, the MSC-exosome and LPS-MSC-exosome groups increased the expression levels of claudin-1 and ZO-1 and decreased levels of caspase-3 and Bax compared to the LPS group. These outcomes aligned with the findings of the *in vivo* tests (264). In another study, Ock et al. (265) developed a model of alcoholic liver injury by exposing hepatocyte organoids (HOs) to alcohol, aiming to evaluate the effectiveness of HOs as a model for liver disease. *Low-density lipoprotein receptor 1* and *sterol regulatory element binding transcription factor 1* were discovered to be elevated, along with BAK, BCL2L1, and caspase 8. Nevertheless, HO lipid accumulation is reduced and hepatocyte apoptosis is inhibited when HOs and adipose-derived MSCs are co-cultured (265). Yang and colleagues discovered that huc-MSC and human adipose tissue-derived MSC-conditioned media may significantly inhibit proliferation and induce apoptosis in the human U251 glioma cell line (266). This suggests that whereas MSCs inhibit apoptosis to mitigate IBD, they may stimulate apoptosis to prevent CRC. Liang and team also found that BMMSC-exosomes may contribute to the prevention of osteomyelitis by stimulating proliferation and osteogenic differentiation and controlling the inflammatory state of bone cells. Staphylococcal protein A (SPA) treated MC3T3-E1 cells to create an *in vitro* osteomyelitis model, and it was found that BCL2 and BCL-XL reduced, whereas BAX increased. Nonetheless, BMMSC-exosome combinations reduced the mRNA level of pro-apoptotic marker BAX in SPA-treated MC3T3-E1 cells while increasing the expression of anti-apoptotic marker genes *(BCL2* and *BCL-XL*) (267).

### 8.2 MSCs and ferroptosis

Wei et al. (34) found that DSS-treated mice experienced diarrhea, bloody stools, weight loss, increased pro-inflammatory cytokines (IL-6, TNF-α, IL-1β), and decreased anti-inflammatory cytokines (IL-10) and occludin and claudin-1. Nevertheless, exosomes produced from human umbilical cord mesenchymal stem cells (hucMSC-exosomes) corrected all of these alterations in mice. Additionally, DMT1, cyclooxygenase 2 (COX2), and ACSL4 showed substantial increases in mRNA expression levels, whereas GPX4 decreased in the DSS group. In contrast, hucMSC-exosome therapy elevated GPX4 and downregulated DMT1, COX2, and ACSL4. The study suggests that hucMSC-Ex plays a protective role in ferroptosis control, potentially preventing it in certain pathways linked to certain genes. In a different study, Li and team revealed that in the lung tissues of burn-induced acute lung injury (ALI) rats, hucMSCs-exosome and Fer-1 (inhibitor of ferroptosis) reduce lung inflammation and increase the levels of the proteins Nrf2 and HO-1. Burn-induced ALI significantly causes ferroptosis, as evidenced by elevated iron and Fe2+ concentrations and reduced SLC7A11 and GPX4 mRNA and protein levels (268). Thus, hucMSCs-exosome may have upregulated SLC7A11 and GPX4 mRNA and protein levels while decreasing iron and Fe2+ concentrations to reduce lung inflammation.

Moreover, Wang and the team found that the administration of LPS caused GPX4 to be down-regulated and ferroptosis-related molecules ACSL4, DMT1, and COX2 to be up-regulated. HucMSC-Ex therapy significantly recovered the depletion of GPX4 while inhibiting the levels of ACSL4, DMT1, and COX2, as observed in the *in vivo* investigation (34). The activation of LPO-related processes in IBD was confirmed by the upregulation of ACSL4 protein and mRNA expression in the LPS-induced inflammatory environment, while hucMSC-Ex therapy led to a decrease in this expression (34). Thus, this confirms hucMSC-Ex’s anti-inflammatory action *in vitro* and its capacity to block LPO, which lowers ferroptosis cell death and heals IBD (34). In a different study, *in vitro*, HucMSCs increased the abundance of SLC7A11 and GPX4 while lowering the expression of genes linked to lipid metabolism, including ACSL4, LPCAT3, and ALOX15, when corpus cavernosum smooth muscle cells are exposed to elevated glucose (269). These demonstrated that HUCMSCs may prevent the ferroptosis signaling pathway in corpus cavernosum smooth muscle cells, reducing erectile dysfunction in diabetes mellitus (269). Zhu et al. (270) found that hUCMSCs prevent type 2 diabetes mice from developing renal ferroptosis.

Wang et al. (271) illustrated the impact of MUC-1 on IEC-6 cell ferroptosis. In IEC-6 cells treated with erastin, it was discovered that when MUC-1 was overexpressed, SLC7A11 and GPX4 increased. Additionally, in cells treated with RSL3, the overexpression of MUC-1 results in the downregulation of ACSL4. This suggests that *MUC-1* might be involved in MSCs’ mode of action in IBD treatment by reducing ferroptosis. MUC1 shields cells from bacterial genotoxins, indicating that cell surface mucins have expanded their functions beyond merely blocking bacterial toxins and are now also defending epithelial cells against xenobiotic toxins (272). It has also been shown that overexpression of *MUC1* attenuates LPS-induced damage of BEAS-2B cells (human alveolar epithelial cell line) *in vitro* (273). While a previous study indicated that *MUC1* could serve as a marker for ferroptosis in UC (274), it has also been demonstrated that the overexpression of MUC1 reduces LPS-induced damage in BEAS-2B cells (a human alveolar epithelial cell line) *in vitro* [258]. This finding supports the research conducted by Wang et al. (271), which revealed that increased levels of MUC1 lead to the downregulation of ACSL4 and the upregulation of GPX4 and SLC7A11.

### 8.3 MSC and pyroptosis

Chang et al. (275) found that the DSS group experienced higher mucosal injury, inflammatory infiltration, diarrhea, bloody stools, and decreased body weight and colon length. Increased levels of NLRP3 and GSDMD protein-positive cells were observed, along with increased levels of IL-1β and IL-18.However, these effects were reversed when MSCs generated from hair follicles (HFMSCs) were administered (275). Similarly, HFMSC exosomes prevented pyroptosis in DSS-treated mice by lowering NLRP3, GSDMD, cleaved caspase-1, and IL-1β proteins, reducing colon damage and the DAI in DSS animals (275). Cai et al. (31)discovered that the IBD mice group exhibited shorter colon length, intestinal injury (abnormality of the colorectal tissue without a typical intestinal gland),and increased pro-inflammatory cytokines and bloody stool; however, these changes were reversed by the administration of hucMSC exosomes. NLRP3 inflammasome-related molecules (NLRP3, ASC, caspase-1, IL-18, and IL-1β) were significantly less expressed in the hucMSC-Ex group than in the IBD group. Mouse peritoneal macrophage IL-1β production was suppressed by hucMSC-derived exosomes even after NLRP3 inflammasome activation. A similar study by Xu and team revealed that the hucMSC-exosome relieves macrophage pyroptosis to ameliorate murine colitis by inhibiting caspase 11 activation and reducing the release of IL-1β, IL-6, and caspase 11.HucMSC exosomes repaired the distorted structure of the colon epithelium (276). These results (31, 276) suggest that hucMSC-exosomes suppressed M1 macrophages, which produce IL-1β.IBD mice’s weight loss, shorter colon, compromised structural integrity of colonic tissues, elevated pyroptosis-related proteins (NLRP3, GSDMD-N, and caspase-1), and proinflammatory cytokines (IL-1β, IL-18, and TNFα) were all reversed by BMSC-derived exosomes, according to another study (277). Bauer et al. (81) found that NLRP3(-/-) mice generated fewer proinflammatory cytokines in colonic tissue and experienced less severe colitis than wild-type mice after oral DSS administration. This implies that NLRP3 expression increases the susceptibility to colitis. Ruan and colleagues have identified pterostilbene analogs as novel NLRP3 inflammasome inhibitors that may be used to treat mice’s DSS-induced colitis (278). Therefore, MSCs and their exosomes may be potential pyroptosis inhibitors.

Though Chang and the team investigated HFMSC exosome prevention of pyroptosis in animal models, similar findings were also observed *in vitro*. Additionally, the study discovered that exosomes significantly inhibited pyroptosis in a dose-dependent manner and somewhat aided *in vitro* regeneration. Western blotting showed that when exosome doses increased, the levels of the proteins NLRP3, GSDMD, cleaved caspase-1, and IL-1β dropped (275). Cai and the team found that HucMSC-derived exosomes improved cell viability *in vitro* following NLRP3 inflammasome activation in THP-1 and MPM cell counting kit-8 assays. They also decreased LDH release, a pyroptotic indicator. THP-1 cells stimulated with LPS and nigericin showed a statistically significant decrease in PI-positive THP-1 cells in the hucMSC-derived exosome-treated group compared to the untreated group, according to flow cytometry analysis. Western blot demonstrated that exosomes produced from hucMSCs prevented GSDMD from being cleaved to the active N-terminal fragment (31). Also, Wang and the team found that exosome treatment produced from BMMSCs decreases proinflammatory cytokines (IL-1β, IL-18, and TNFα), ROS levels, and pyroptosis (NLRP3, GSDMD-N, and caspase-1) in mouse small IECs (mIECs) treated with LPS in a manner that is dependent on miR-539-5p (277). In a different study, the hucMSC-exosome has been found to target METTL14, effectively enhancing nucleus pulposus (NP) cell viability and protecting them against pyroptosis (279). Pei et al. (280) also found that exosomes generated from BMSCs prevented heat-stroke-induced pyroptosis in human umbilical vein endothelial cells.

### 8.4 MSCs and necroptosis

**Figure 3** previously illustrated the identification of STAT1 and JAK1 as contributors to the development of necroptosis. Cao et al. (281) revealed that BMMSC-derived EVs significantly reduced the phosphorylation of JAK1 and STAT1 in DSS-induced UC. In a different study, adipose-derived MSCs and EVs treatment decreased JAK1 in DSS-induced colitis (282). These suggest that MSCs-EVs may help prevent necroptosis in IBD.

Although research on MSCs and their exosome modulation of necroptosis in IBD is sparse, previous studies have demonstrated that MSCs can regulate necroptosis. As a result, the involvement of MSCs and their EVs in controlling necroptosis in IBD is a developing topic that needs to be investigated. Yuan et al. (283) discovered that human-induced pluripotent stem cell-derived mesenchymal stromal cells (hiPSC-MSCs)-EVs can reduce renal ischemia-reperfusion damage by activating sphingosine kinase 1 and inhibiting necroptosis. There was no discernible difference between the administration of hiPSC-MSCs-EVs alone and the necroptosis inhibitor necrostatin-1 (Nec-1) pretreatment. Studies have proven Nec-1’s ability to suppress necroptosis in IBD (118, 120, 284). Therefore, hiPSC-MSCs-EVs may regulate necroptosis molecules (RIPK1/3) and MLKL in IBD treatment. In pigs suffering from metabolic syndrome (metS) and renal artery stenosis (RAS), MSCs derived from adipose tissue and their EV improve the function of stenotic kidneys and lessen tissue damage (285). EV effectively preserved renal cellular integrity, while MSCs successfully protected the microcirculation (285). The metS + RAS group exhibited higher levels of RIPK1 and RIPK3, but the EV from MSC decreased these levels (285). Similar findings have been reported in severe acute pancreatitis (SAP), where miR-9 in BMMSCs decreased RIPK1, RIPK3, and p-MLKL (286). Notably, studies have shown that MSCs and their EVs can improve other diseases and possibly IBD. As a result, MSCs and their EVs may be able to modulate necroptosis during IBD therapy. We should further investigate this growing field.

### 8.5 Autophagy modulation by MSCs

Li et al. (287) found that mice treated with DSS showed infiltration of inflammatory cells, damaged colonic mucosa, missing glands, and decreased colon length and body weight. Nevertheless, all of these alterations were restored by hypoxia-preconditioned HF-MSC-derived exosomes (Hy-Ex)(i.e., reduced infiltration of inflammatory cells, improved colonic mucosa damage, and so on). Western blotting showed that after Hy-Exos therapy, the expression of p-mTOR/mTOR, p-AKT/AKT, and p-PI3K/PI3K decreased following an initial increase in the UC group (287). The autophagy process is mainly regulated by the kinase mTOR, which is affected by cellular stress, growth factors, and starvation (288). mTOR kinase regulates autophagy, while the PI3K/AKT survival pathway influences it indirectly (288). Therefore, MSCs’ ability to regulate the PI3K/AKT/mTOR route may be able to regulate autophagy. Studies have shown increased mTOR expressions in IBD (148, 150, 151). Therefore, Hy-Ex downregulating p-mTOR/mTOR may make it a potential autophagy regulator. In a different study, inhibition of the PI3K/AKT/mTOR pathway reduces Piezo1 inhibition of chondrocyte autophagy (289), implying that PI3K/AKT/mTOR activation increases Piezo1 inhibition of chondrocyte autophagy. MSC-Exo-mediated KLF3-AS1 repressed autophagy and apoptosis of chondrocytes by activating PI3K/Akt/mTOR signaling pathway (290). Additionally, research shows increased P62, mTOR, and p-mTOR levels in experimental colitis, while LC3B and ATG16L1 levels are reduced. However, rapamycin intervention improved colonic pathology in mice, reducing disease activity index score (148). Furthermore, the rapamycin group is revealed to have a significant abundance of *Lactobacillus reuteri (L. reuteri)* (148). Interestingly, a mouse model has shown that stem-cell-loaded hydrogel microcapsules (SC-HM) can enhance MSC (huc blood MSC) survival and retention in the stomach, promote tissue restoration, decrease colonic macrophage invasion, and significantly reduce the severity of IBD (291). SC-HM also enhanced *L. reuteri* in the inflammatory colon (291). These results show that SC-HM is a viable therapeutic option for IBD (291). *L. reuteri* aids in repairing intestinal diseases by protecting the gut barrier, inhibiting oxidative stress, regulating gut microbiota metabolism, and suppressing inflammation and immunological responses (292). As a result, MSCs may be able to modulate autophagy in IBD by regulating the gut flora.

**Figure 6:**
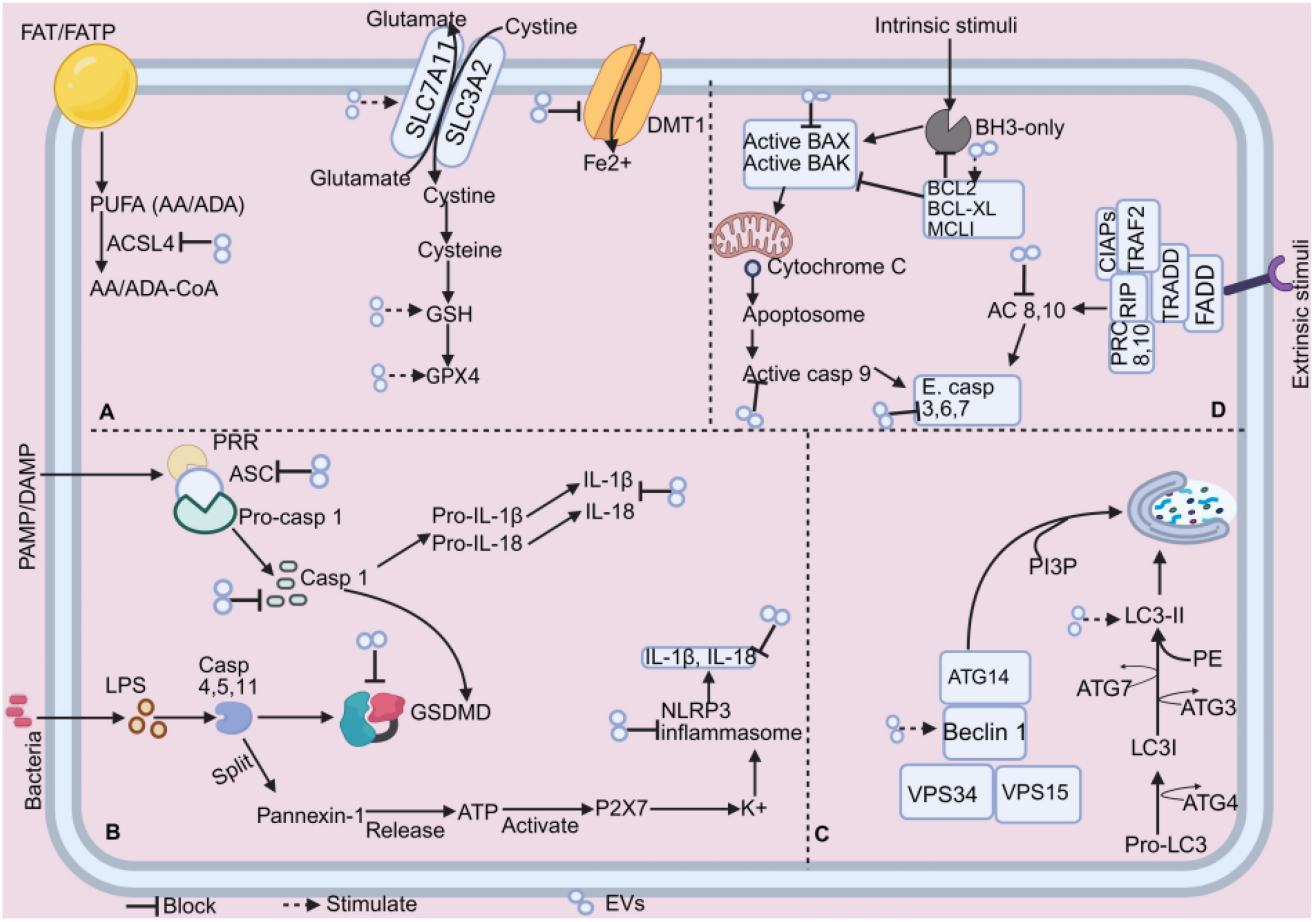
Summary of MSC + EV regulation on key cell death markers of IBD. The figure summarizes how MSC + EV regulates specific cell death markers to prevent or activate subsequent downstream signaling, as shown in **Figures 1**, **2**, **3**, **4**, **5**. **(A)** Ferroptosis, **(B)** Pyroptosis, **(C)** Autophagy **(D)** Apoptosis. ACSL4, long-chain acyl-coenzyme A synthase 4; ASC, apoptosis-associated speck-like protein containing card; ATG, autophagy-related gene; BAX, bcl-2 associated x protein; BH3, bcl2 homology domain 3; Casp, caspase; CIAPs, cellular inhibitor of apoptosis proteins; DAMP, damage-associated molecular patterns; DMT1, divalent metal ion transporter 1; E, effector; FADD, fas-associated death domain protein; GPX4, glutathione peroxidase-4; GSDMD, gasdermin d; GSH, glutathione; IL, interleukin; LC3, microtubule-associated protein 1 light chain 3; NLRP3, nod-like receptor pyrin domain-containing protein 3; PAMP, pathogen-associated molecular patterns; PI3P, phosphatidylinositol 3-phosphate; PRC, procaspase; PRR, pattern-recognition receptor; PUFA, polyunsaturated fatty acid; TRADD, TNF receptor-associated death domain protein; TRAF2, TNF receptor-associated factor 2; VPS, vacuolar protein sorting.

**Figure 7:**
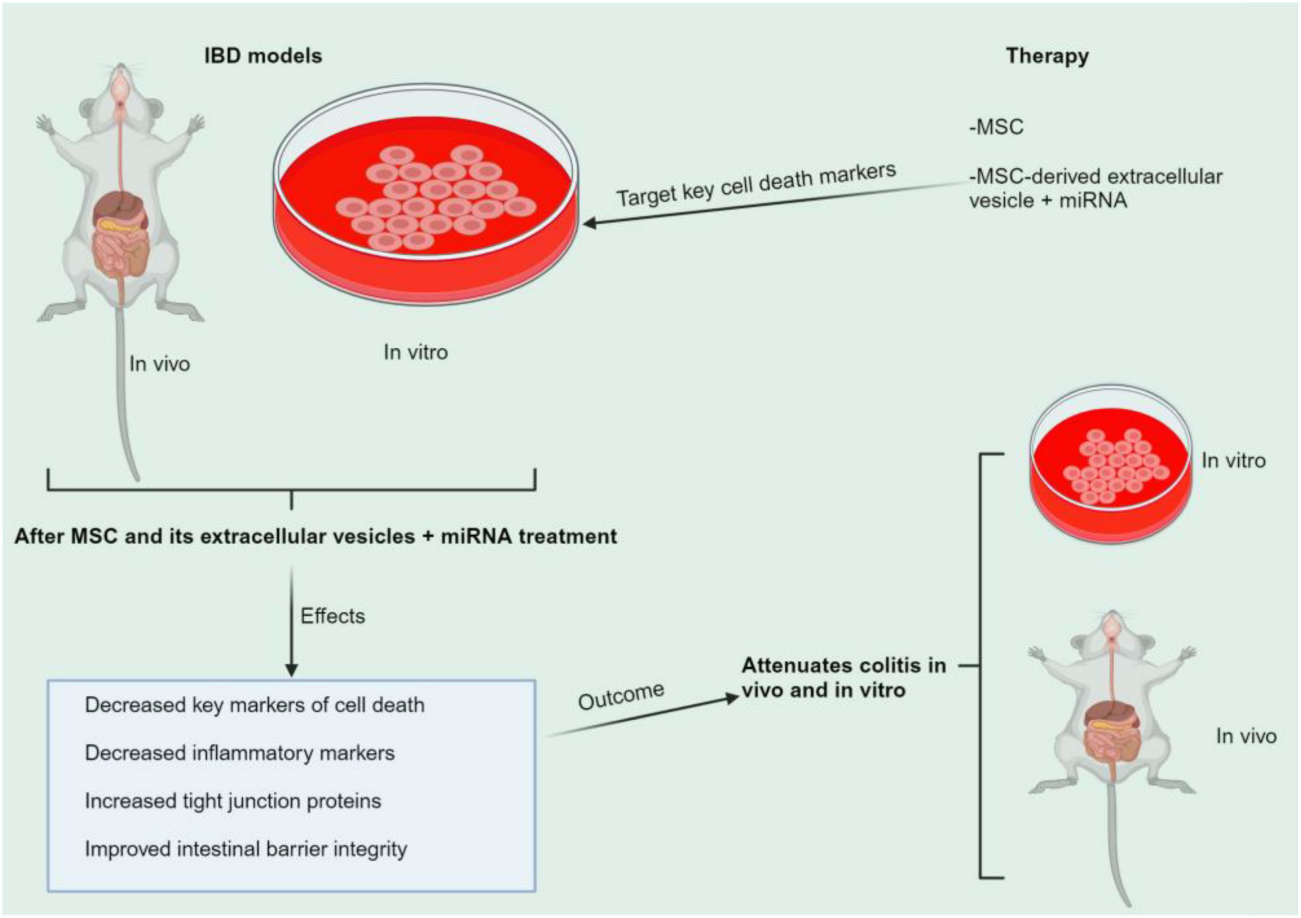
Summary of the role of MSCs in targeting cell death markers of IBD. MSCs and their mediators target cell death indicators *in vitro* and *in vivo* to reduce inflammatory markers, disrupt tight junction proteins, and improve intestinal barrier integrity, thereby alleviating colitis. IBD, inflammatory bowel disease; miRNA, microRNA; MSC, mesenchymal stem cell.

In MODE-K cells, Hy-Ex reversed the LPS-induced reductions in Beclin1 and LC3II/I expression and raised in p62, HSP60, and TOMM20 expression (287). These show that Hy-Exos may regulate autophagy in IBD. Research has demonstrated decreased expressions of LC3II/I and beclin 1 in colitis (148, 149). Hence, Hy-Ex upregulates LC3II/I and beclin 1, making it a potential candidate for preventing colitis. MScs’ function in targeting IBD cell death markers is summarized in **Figures 6**, **7**, and **Table 1**.

**Table 1:** Summary of MSC regulation of cell death markers in IBD.

Types of MSCs	Model used in MSC treatment	Impact on cell death markers/pathways	Impact on inflammatory marker expression	Impact on tight junction protein expressions	Outcome	References
Apoptosis						
Adipose						
Filtrated murine adipose-derived MSC lysate	*In vivo*	↓Cleaved caspase-3-positive cells ↓Apoptotic nuclear cells	↓TNF-α, ↓IL-6 ↓PAI-1	↑Claudin-2 ↑Occludin	Improves colitis	(253)
ASCs + sulfasalazine therapy	*In vivo*	↓Bax ↑BCL2	↓TNF-α ↓IL-1 ↓IL-6 ↓IL-17 ↑IL-10 ↑TGF-β	–	Slow the development of colitis	(259)
Bone marrow						
Heme oxygenase-1-modified bone marrow MSC-derived exosome and its miR-200b	*In vitro*	↓Cleaved caspase-3 ↓BAX/BCL2 ratio	↓HMGB3	↑Zona Occludens 1	Reduces intestinal injuries	(262)
Bone marrow-derived MSC conditioned medium	*In vivo*	↓BAX, ↓Caspase 3 ↓Cleaved caspase 3 ↑BCL2	↓IL-1β ↓TNF-α ↓IL-6	↑Zona Occludens 1	Repairs damage to colonic epithelial cells by preventing apoptosis.	(255)
Bone marrow-derived MSC-derived EVs containing miR-378a-3p	*In vitro*	↓GATA2/AQP4/PPAR‐α	–	–	Minimizes M064 cells’ LPS-induced apoptosis	(263)
Bone marrow-derived MSC-derived EVs	*In vivo*	↓Cleaved caspase-3 ↓Cleaved caspase-8 ↓Cleaved caspase-9	↓IL-1β ↓TNF-α ↓COX-2	–	Reversal of experimental colitis	(254)
MSC-derived exosomal miR-181a	*In vivo*/*in vitro*	↓Caspase-3 ↓Bax ↑Bcl-2	↓TNF-α ↓IL-6 ↓IL-1β ↓IL-17 ↓IL-18	↑Claudin-1, ↑ZO-1	Prevents colitis via transferring miR-181a	(264)
Embryonic						
MSCs from human embryonic stem cells (T-MSCs)	*In vivo*, *in vitro*	↓Annexin V (+) 7-AAD (–) apoptotic cells	↓CXCL1, ↓CXCL2, ↓IL-6 ↓ MCP-1	–	Reduced colitis in mice by increasing the amount of IGF-1 in the blood.	(257)
Ferroptosis						
Umbilical cord						
Human umbilical cord MSC	*In vivo*/*in vitro*	↓*ACSL4* *↑SLC7A11* *↑GPX4* (The DSS-MSC group shows high *MUC-1* expression and high *MUC-1* leads to the above expression levels)	↓IL-1β ↓TNF-α ↓IL-6	-	Decrease cell death and improve IBD healing.	(271)
Human umbilical cord MSC-derived exosomes and its miR-129-5p	*In vivo*/*in vitro*	↓ACSL4 ↓DMT1 ↑GPX4 ↑GSH	↓IL-6 ↓TNF-α ↓IL-1β	↑Occludin, ↑Claudin-1	Lessens intestinal inflammation and repairs the harm	(34)
Pyroptosis						
Umbilical cord						
Human umbilical cord MSC-derived exosomes and its miR-378a-5p	*In vivo*/*in vitro*	↓ NLRP3 ↓Caspase-1 cleavage ↓ASC ↓GSDMD cleavage ↓IL-1β ↓IL-18	↓IL-6 ↓TNF-α ↑IL-10 ↓IL-1β ↓IL-18	–	Guard against colitis	(31)
Human umbilical cord MSC-secreted exosomes	*In vivo*/*in vitro*	↓Caspase 11 ↓Caspase 4 ↓GSDMD	↓IL-1β ↓IL-6	–	Improves colitis	(276)
Hair follicle (HF)						
HFMSCs	*In vivo*/*in vitro*	↓ NLRP3 ↓GSDMD ↓Cleaved caspase-1 ↓IL-1*β* ↓IL-18	↓IL-1*β* ↓IL-18	–	Prevent pyroptosis to lessen UC brought on by DSS	(275)
Bone marrow						
Bone marrow-derived MSC-derived exosome and its miR-539-5p	*In vivo*/*in vitro*	↓NLRP3 ↓GSDMD-N ↓Caspase-1	↓IL-1β ↓IL-18 ↓TNF-α	–	Impedes the development of IBD	(277)
Autophagy						
Hair follicle (HF)						
HF-MSC-derived exosomes preconditioned by hypoxia	*In vivo*/*in vitro*	↑Beclin1 ↑LC3II/I	↓IL-1β ↓TNF-α ↑IL-4 ↑IL-10	↑Claudin1 ↑Occludin	Hy-Exos improve mitophagy and reduce UC	(287)

ACSL4, long-chain acyl-coenzyme A synthase 4; AQP4, aquaporin-4; ASC, apoptosis-associated speck-like protein containing card; BAX, bcl-2 associated x protein; BCL2, b-cell lymphoma-2; COX, cyclooxygenase; DMT1, divalent metal ion transporter 1; DSS, dextran sulphate sodium; EV, extracellular vesicle; GATA2, GATA-binding protein 2; GPX4, glutathione peroxidase-4; GSDMD, gasdermin d; GSH, glutathione; HMGB3, high mobility group box 3; IBD, inflammatory bowel disease; IGF-1, insulin-like growth factor-1; IL, interleukin; LC3, microtubule-associated protein 1 light chain 3; LPS, lipopolysaccharides; miR, microRNA; MSC, mesenchymal stem cell; NLRP3, nod-like receptor pyrin domain-containing protein 3; PPAR-α, peroxisome proliferator-activated receptor-α; SLC, solute carrier family; TNF-α, tumor necrosis factor alpha; UC, ulcerative colitis. The dash sign (-) indicates that although tight junction proteins were not determined, intestinal damage was assessed using other methods, and interventions reduced the damage.

## 9 Emerging research and potential therapeutic avenues

### 9.1 Clinical trials involving MSCs for IBD treatment

Several clinical trials have also demonstrated the usefulness of employing MSC to treat IBD.

For instance, a clinical trial found that patients with CD on a steady steroid dosage can greatly benefit and safely receive UC-MSC treatment (293). In this trial, 82 individuals with a CD diagnosis who had been on steroid maintenance medication for longer than six months were included. Every week, four times, patients in the UC-MSC group got an injection of 1×10^6^ cells/kg. Patients were injected with 2,500 IU of low-molecular-weight heparin daily for three days to prevent thrombosis before surgery and then monitored for three, six, nine, and twelve months. The Harvey-Bradshaw index (HBI), corticosteroid dosage, adverse events, and CD activity index (CDAI) were evaluated at every follow-up visit (293). Colonoscopy results showed that the UC-MSC group’s CDAI decreased from 9.2 ± 1.5 before therapy to 3.4 ± 1.2 at the 12-month follow-up. The UC-MSC group experienced a decrease in CDAI, HBI, and corticosteroid dosage of 62.5 ± 23.2, 3.4 ± 1.2, and 4.2 ± 0.84 mg/day, respectively, while the control group experienced a drop of 23.6 ± 12.4, 1.2 ± 0.58, and 1.2 ± 0.35 mg/day (293). Four patients experienced fever after cell infusion, which subsided after symptomatic therapy, and seven patients experienced nine upper respiratory tract infection episodes within six months. There were no significant adverse effects noted (293). It is well established that MSCs can inhibit immune responses and have therapeutic promise for establishing transplant tolerance (294). This property may have caused infections and fever due to immune suppression. As a result, MSC might be more secure and useful in a medical context. A different trial showed that in patients with type 2 diabetes, administering UC-MSCs by intravenous infusion is a safe and efficient method that may lessen the need for exogenous insulin and improve insulin resistance (295). Transplanting UC-MSCs may be a viable treatment for type 2 diabetes (295). Zang et al. (296) revealed that the intravenous infusion of UC-MSCs is a successful strategy for reducing glycemic fluctuation and the time in range in type 2 diabetes. Bartolucci and the team found that in patients with stable heart failure and a lower ejection fraction receiving the best medical care, intravenous UC-MSC infusion proved safe (297). Patients treated with UC-MSCs showed improvements in their quality of life, functional status, and left ventricular function (297). Lanzoni et al. (298) found that UC-MSC infusions are safe for COVID-19 ARDS treatment, reducing inflammatory cytokines at day six and increasing patient survival rates.

Another study investigated the efficacy of allogeneic MSCs in patients with luminal CD (299). On day 42, the mean CDAI score for the 15 patients who finished the study dropped from 370 to 203. After every MSC infusion, the average CDAI scores dropped (299). Eight patients experienced clinical remission, while twelve experienced a clinical response. Seven patients (47%), whose mean CDEIS scores dropped from 21.5 to 11.0, experienced endoscopic improvement. Patients’ quality of life (QoL) was enhanced by MSC (299). IBD symptoms can include diarrhea, stomach pain, gastrointestinal bleeding, weight loss, malnourishment, and exhaustion. These symptoms may lead to lifestyle restrictions and significant psychosocial effects, ultimately impacting the QoL for those affected (300). Adults and children with IBD have lower QoL than healthy people, according to a meta-analysis review (301). Graff et al. (302) also found that those with active IBD experienced reduced social support, well-being, mastery, and disease-specific QoL, as well as higher levels of discomfort, health anxiety, and perceived stress. Therefore, MSC may enhance symptoms of IBD while also improving QoL.

Panés et al. (303) looked at the effectiveness and safety of using expanded, allogeneic, adipose-derived stem cells (Cx601) for CD patients’ treatment-refractory complicated perianal fistulas. Out of the 212 patients that took part, 107 were given Cx601 and 105 were given a placebo. The Cx601 group showed a higher percentage of patients experiencing total remission compared to the placebo group (303). Complex perianal fistulas in CD patients who did not react to biological, conventional, or both types of therapy can be safely and effectively treated with Cx601 (303). A related trial involving 212 individuals found that when Cx601 was administered once to patients with complex perianal fistulas who were not responding to treatment for CD, its effectiveness lasted for up to a year (304). Therefore, for difficult perianal fistulas, Cx601 offers a unique and least invasive substitute that might lessen the need for surgery or systemic immunosuppression (304). In a trial conducted by Furukawa and the team, it was found that 22 patients who received darvadstrocel (a suspension of expanded, allogeneic, adipose-derived MSCs) and completed the 52-week follow-up between March 6, 2019, and February 1, 2021, achieved a combined remission (magnetic resonance imaging-confirmed absence of collections more than 2 cm and clinically verified closure of all treated external holes that were draining during screening) rate of 59.1% by week 24 (305). At week 52, the effect persisted, with 68.2% of patients experiencing combined remission (305). Perianal illness is among the most incapacitating signs of CD and rarely in cases of UC (306, 307). Additionally, it might be challenging to treat pouch CD patients who develop perianal illness (such as fistula), which occasionally calls for pouch excision (308). Tan and colleagues discovered that while fistulas do not raise the risk of IBD, CD does inadvertently raise the chance of fistulas more than UC (309). De la Poza et al. (310) also found that surgical therapy is the most effective treatment for genital fistulas, which are a major issue for female CD patients. Consequently, MSC might be a possible remedy for perianal conditions (like fistula).A different trial also showed that in patients with COVID-19, nebulized exosomes made from human adipose-derived MSC (haMSC-Ex) enhanced computed tomography image scores and clinical symptoms (311). The haMSC-Ex nebulization was well tolerated by all COVID-19 patients, and neither the nebulization nor the immediate post-nebulization interval showed any signs of clinical instability or predicted adverse events (311). **Table 2** highlights the use of MSCs in IBD in clinical trials.

**Table 2:** Summary of MSCs’ success in clinical trials.

Clinical trial ID	Type of clinical trial	Condition	Intervention	Study location (s)	Outcome	Reference
NCT02445547	Prospective, randomized, controlled, open-label	CD	Umbilical cord MSCs	China	Decreased CDAI, HBI, and corticosteroid dosage	(293)
NCT01090817	Open-label, multicenter, Australian, nonrandomized	Luminal CD	Allogeneic MSCs	Australia	Reduced CDAI and CDEIS scores, endoscopic improvement	(299)
NCT01541579	Phase 3 randomised, double-blind controlled trial	Complex perianal fistulas in patients with CD	Allogeneic, expanded, adipose-derived MSC	Seven European countries and Israel	*Combined remission	(303)
NCT01541579	Randomized placebo-controlled	CD and perianal fistulas	Allogeneic expanded adipose-derived stem cells	Europe and Israel	*Combined remission	(304)
NCT03706456	Phase 3, multicentre, open-label, uncontrolled	Complex perianal fistulas in patients with CD.	Darvadstrocel, a suspension of expanded, allogeneic, adipose-derived, MSC	Japan	*Combined remission	(305)

*clinical assessment of closure of all treated external openings that were draining at baseline, and absence of collections >2 cm of the treated perianal fistulas confirmed by masked central MRI). CDAI, Crohn’s disease activity index; CDEIS, CD endoscopic index of severity; HBI, harvey-Bradshaw index; MRI, magnetic resonance imaging; MSC, mesenchymal stem cell.

### 9.2 Combination therapy using MSC, exosome, and IBD treatment drugs

Numerous studies have shown the effectiveness of conventional medicines in mitigating IBD. Additionally, a retrospective investigation discovered that 5-ASA was frequently utilized as a long-term CD therapy, and the usage of CD-related healthcare resources declined significantly in the year following the implementation of 5-ASA (312). According to Otkur et al. (313), aminosalicylates can prevent DSS-induced colitis by targeting GPR35. Other studies have shown the efficacy of aminosalicylates in treating IBD (314, 315). Moreover, the use of corticosteroids (316–318), immunomodulators (319–321), small molecule inhibitors (322, 323), and biologics (324–328) has also shown efficacy in IBD. Despite their therapeutic efficacy, some patients may not respond because of side effects and high costs (329, 330). However, the combination of these IBD medicines with MSCs and exosomes may be crucial in IBD treatment since these drugs have shown promise in reducing IBD alone. A study found that combining ASCs with traditional IBD treatment may be a far more effective way to delay the disease’s progression by lowering inflammatory and apoptotic indicators than either treatment alone (259). Interestingly, another study found that combination therapy with 5-ASA and MSC increases apoptosis and inflammation. The combined 5-ASA and MSC treatment had a detrimental impact on UC mice (331). 5-ASA can enhance inflammatory factors, cause cell death (apoptosis), and inhibit MSC development. 5-ASA considerably decreased the colon’s MSC content (331). As a result, combined therapy with MSC and IBD medicine may not be practical at this time, and further research is required to investigate this area due to limited data. While more research is being conducted, clinicians should exercise caution when considering combination therapy with MSCs, exosomes, and IBD drugs. Other IBD medicines with MSC should also be explored.

## 10 Challenges and limitations

The lack of confidence in MSC therapy stems from inconsistent pre-clinical and clinical outcomes and the mismatch between predicted and actual efficacy in various illnesses (332). Beyond safety, clinical effectiveness remains very diverse and contentious, with no evidence of a molecular explanation and conflicting therapeutic advantages (333). For instance, several clinical trials on MSC treatments in IBD (CD) reported fever (293), anal abscess and proctalgia (303), worsening of CD, diarrhea, increased blood bilirubin (305), and anal abscess/fistula (304). The trials suggest that while MSCs are safe and effective, their adverse effects may pose practical challenges. Interestingly, one study identified a major adverse event (2 dysplasia-related lesions) (299). Nonetheless, the study stated that MSCs most likely did not cause this. Research has indicated that MSCs might encourage the growth of tumors. For instance, the proangiogenic factors released by cancer cells to promote angiogenesis and tumor development may be enriched by IL-6 produced by MSCs; addressing this relationship may result in new preventative and therapeutic approaches (334). IL-6, secreted by MSCs, stimulates the growth of colorectal tumor-initiating cells and encourages tumor development through STAT3 signaling (335). In a different investigation, MSCs improved the capacity of lung cancer cells A549 and CL1–5 to grow tumors in immunocompromised mice (336). Another study has also demonstrated that MSC treatment has a protective effect on tumor development. For instance, Hu and colleagues discovered that late administration of MSCs promotes colitis-associated cancer (CAC) development, while early administration prevents CAC incidence (337). On the contrary, issues with EVs, the mediators of MSCs, have yet to be reported in IBD. As a result, further clinical studies with EVs should be considered. EVs, the mediators of MSCs, could be the new era of treating IBD-associated cell death.

Several limitations have been identified with the use of MSCs. Disease results vary significantly depending on the type of mesenchymal cells used, how the cells are preconditioned, how often treatments are administered, and how they are administered. Thus, a more thorough study of MSCs is required (255). According to the review, different MSC sources and conditioning methods are employed, which may influence the result of the MSCs. For instance, Nishikawa and colleagues found that while cell-free lysate injection produced a comparable improvement to the previously documented MSC treatment, the mechanism by which pleiotropic factors ameliorate DSS-induced colitis remains unknown. Also, what precisely makes up FADSTL and the process that underlies their apparent effectiveness is uncertain. Further research is needed to comprehend the mechanisms behind its anti-inflammatory and anti-apoptotic properties (253). Another study found that the mechanism of miR-378a-5p controlling NLRP3 is non-specific, highlighting the need for more research to gain clarification (31).

## 11 Conclusion and future directions

MSCs and their mediators have been shown to regulate cell death pathways in various diseases, including IBD. MSCs alleviate barrier defects and decrease immune cell infiltrations and inflammatory cytokine release. However, side effects may pose challenges in clinical settings. Clinical investigations show insufficient evidence to relate EVs to cell death in IBD, and EV use has not been associated with any preclinical negative effects. The diversity of extracellular vesicles (EVs) (338, 339) and the minimal uniformity regarding the methodologies for their isolation, purification, and characterization (340) make the application of EVs in the clinical setting challenging. Additionally, the wide range of methods utilizing unique biochemical characteristics of EVs and the lack of standardized protocols make data interpretation extremely challenging (341). The review’s findings showed evidence that may impede clinical research using EVs in IBD. For instance, regarding the appropriate dosage for EV use, different research studies have used varying amounts of EVs in colitis treatment; hence, there is no standardized dose when translating to clinical trials involving humans. Additionally, colitis induction to mimic the human model is created using different concentrations, making standardization of this model with EV treatment challenging for clinical studies. Frequent injections with EVs in animal models may also pose a challenge during clinical studies. Using different animal species for colitis model, different sources of EVs, and frequency of administration of EVs may also affect translation to clinical settings. Although these preclinical studies have shown the potential to explore the modulatory mechanism of EVs in IBD, the complete understanding of the mechanism in human IBD is lacking, therefore a study suggested the exploration of EVs in human model to better understand its mechanism (287). Notwithstanding these difficulties, EVs might one day be used to diagnose IBD, as is the case with CRC (342), given that IBD is a risk factor for CRC. Studies should focus on the standardization of protocols and the uniformity of EV extraction for IBD experiments, other routes of EV administration (oral) should be explored, and efforts to research larger sample sizes in clinical studies should be encouraged. Preclinical and clinical research should focus on MSCS and other cell death mechanisms with less evidence, such as cuproptosis. Cuproptosis, a type of cell death involving copper ions being delivered to lipoylated TCA cycle proteins, is a widely researched topic in cancer research. However, it is also linked to harmful processes like oxidative stress, apoptosis, and inflammation (343, 344). Studies have also shown that cuproptosis genes may serve as biomarkers for IBD, and these genes have been associated with immune cell infiltration regulation in IBD (345–347). Nevertheless, the direct mechanism of MSCs targeting cuproptosis is novel and needs further exploration since cuproptosis may be implicated in the pathogenesis of IBD. Moreover, as cuproptosis represents a novel form of cellular death and has been insufficiently studied, further research will enhance the limited understanding of cuproptosis, offer innovative therapeutic strategies, elucidate disease variations, and furnish methodologies for assessing and identifying cell death pathways. Further exploration of MSCs and exosomes with other IBD medicines in cell death is warranted.
